# Imatinib decreases germ cell survival and germline stem cell proliferation in rodent testis ex vivo and in vitro

**DOI:** 10.1111/andr.13777

**Published:** 2024-10-18

**Authors:** Anna Eggert, Sini Laasanen, Mirja Nurmio, Aida Wahlgren, Kirsi Jahnukainen, Kim Eerola, Miisael Nieminen, Opeyemi Olotu, Noora Kotaja, Juho‐Antti Mäkelä, Jorma Toppari

**Affiliations:** ^1^ Research Centre for Integrative Physiology and Pharmacology Institute of Biomedicine University of Turku Turku Finland; ^2^ Tyks Acute Turku University Hospital Turku Finland; ^3^ Department of Women's and Children's Health Karolinska Institutet and University Hospital Solna Sweden; ^4^ Division of Hematology‐Oncology and Stem Cell Transplantation New Children's Hospital Pediatric Research Center University of Helsinki and Helsinki University Hospital Helsinki Finland; ^5^ Department of Genomics Turku University Hospital Laboratories Turku Finland; ^6^ Department of Pediatrics Turku University Hospital Turku Finland; ^7^ Centre for Population Health Research University of Turku and Turku University Hospital Turku Finland; ^8^ InFLAMES Flagship Research Centre University of Turku and Åbo Akademi University Turku Finland

**Keywords:** dasatinib, imatinib, male fertility, male germline stem cells, spermatogenesis, testis

## Abstract

**Background:**

Imatinib and dasatinib are tyrosine kinase inhibitors (TKIs) increasingly used to treat several diseases in both children and adults at fertile age. We have previously shown that imatinib has adverse effects on developing testis, and imatinib‐treated male patients have been reported to have reduced sperm counts. However, the cellular level effects of imatinib and dasatinib on adult male germ cells and germline stem cells (mGSCs) have not been thoroughly investigated.

**Objectives:**

To analyze whether imatinib or dasatinib exposure ex vivo and in vitro is harmful to adult male rodent germ cells and mGSCs.

**Materials and methods:**

Seminiferous tubule segments of adult male mouse or rat were cultured in the presence or the absence of imatinib or dasatinib. Stage‐specific effects were monitored by ^3^H‐thymidine incorporation assay (DNA synthesis), immunohistochemistry (cleaved Caspase‐3; apoptosis), immunofluorescence (KI67, GFRα1, STRA8, c‐KIT, LIN28A; proliferation and spermatogonial differentiation) and flow cytometry (Hoechst). Mouse mGSCs were exposed to imatinib and dasatinib to study proliferation, apoptosis, and differentiation.

**Results:**

Imatinib decreased stage‐specific DNA synthesis, and induced apoptosis in cultured rat seminiferous tubule segments. Imatinib also had an adverse effect on mGSC proliferation both in vitro and ex vivo, but did not induce cell death in cultured mGSCs. Imatinib did not impinge on induction of spermatogonial differentiation but suppressed c‐KIT expression in nascent differentiating spermatogonia, providing a plausible mechanism for its pro‐apoptotic function in spermatogenic cells. Clinically relevant doses of dasatinib did not induce apoptosis in seminiferous tubules but decreased mGSC colony growth in vitro.

**Conclusions:**

Imatinib exposure ex vivo and in vitro impinges on male rodent germ cell proliferation and survival. The plausible mechanism in spermatogenic cells is the inhibition of SCF/c‐KIT signaling, and reduced expression of c‐KIT. Dasatinib did not show significant adverse effects with clinical doses ex vivo but inhibited mGSC colony growth in vitro.

## 1 INTRODUCTION

Tyrosine kinases (TKs) have important roles in cell growth, proliferation, metabolism, and cell death pathways.^1^ Therefore, inhibition of protein tyrosine kinases is one of the leading approaches to targeted cancer therapy.^1^ Imatinib mesylate is a first‐generation small‐molecule protein tyrosine kinase inhibitor (TKI), and it is widely used as a treatment against, for example, chronic myeloid leukemia (CML), Philadelphia chromosome‐positive acute lymphoblastic leukemia (ALL, Ph+), and gastrointestinal stromal tumors (GIST).^2^ Imatinib is a potent inhibitor of BCR‐ABL, platelet‐derived growth factor receptor (PDGFR), ARG, and c‐KIT TKs.^2^, ^3^ Most patients treated with imatinib live longer than 10 years after the onset of treatment.^2^, ^4^, ^5^ Imatinib treatment is used to control and prevent the disease from progressing, and therefore chronically administered.^6^ Notably, the clinical utilization of imatinib is increasing. Imatinib has, for instance, shown promising results in the prevention of inflammatory diseases.^7^, ^8^, ^9^ Recent results from preclinical studies suggest that imatinib could also be beneficial in preventing or reversing the onset of type 1 diabetes,^10^, ^11^ which has also been tested in clinical trials^12^ and most recently in a pediatric patient with type 1 diabetes.^13^

Imatinib resistance and intolerance have been long recognized.^14^, ^15^ Dasatinib is a second‐generation TKI and it inhibits BCR‐ABL, the SRC family kinases (SRC, LCK, HCK, YES, FYN, FGR, BLK, LYN, FYN‐related), receptor tyrosine kinases (c‐KIT, PDGFR, DDR1 and 2, c‐FMS, ephrin receptors), and TEC family kinases (TEC and BTK).^16^ Dasatinib is used for the BCR–ABL‐driven diseases, such as CML and Philadelphia‐chromosome‐positive acute lymphoblastic leukemia (Ph+ ALL) especially when imatinib resistance or intolerance occurs.^16^ In preclinical and clinical studies, dasatinib has shown to be more potent BCR–ABL inhibitor than imatinib.^17^, ^18^ Dasatinib is also considered a senolytic drug and in combination with flavonol quercetin it has been tested in clinical trials aiming to eliminate senescent cells and therefore to treat diseases where cell senescence is involved.^19^ Like imatinib, dasatinib is also used both in children and fertile adults.^16^

Spermatogenesis is a highly efficient differentiation process taking place in seminiferous tubules in the testis producing tens to hundreds of millions of sperm per day.^20^ Two types of cells are found inside the seminiferous tubules: germ cells, including spermatogenic cells and male germline stem cells (mGSCs), and somatic Sertoli cells, which nurture and guard the germline. mGSCs locate on the basement membrane of seminiferous tubules and they maintain life‐long spermatogenesis and male fertility by undergoing self‐renewal and differentiation.^21^ The differentiating progeny first expands mitotically, then enters and completes meiosis, and finally undergoes spermiogenesis to produce sperm.^20^ We have previously shown that imatinib interferes with several maturation processes in the prepubertal rat testis, including gonocyte migration, testicular growth, formation of the mGSC pool, and proliferation of differentiating type A spermatogonia.^22^ Despite extensive acute effects, adult male rats treated prepubertally with imatinib displayed qualitatively and quantitatively normal spermatogenesis,^22^ but sired slightly smaller litters,^23^ suggesting existence of latent effects from exposure to imatinib in childhood. Conversely, long‐term treatment of adult male mice with clinical doses of imatinib leads to plummeting sperm counts, suggesting that TKs targeted by imatinib are crucial for spermatogenesis.^24^ In imatinib‐treated patients, reduced sperm count and semen quality have also been reported.^25^, ^26^, ^27^, ^28^ Interestingly, there are some indications that the effects of dasatinib on spermatogenesis are milder than those of imatinib.^29^ Garcia et al. studied the effects of dasatinib and quercetin on male mice in vivo and they did not observe adverse effects on mouse spermatogenesis after 5‐month exposure.^30^ However, cell‐level effects of imatinib and dasatinib on spermatogenic cells and mGSCs have remained under‐studied.

While the cancerous targets of imatinib and dasatinib are perhaps not significant for spermatogenesis, there are at least two target receptor TKs of imatinib and dasatinib with well‐established roles in testicular development, physiology, and function: c‐KIT and PDGFR. c‐KIT is expressed in differentiating spermatogonia, preleptotene spermatocytes and Leydig cells; and in a truncated form in round spermatids.^31^, ^32^ Stem cell factor (SCF) signaling through c‐KIT receptor is essential for spermatogonial proliferation and survival.^33^, ^34^, ^35^, ^36^ Also, PDGF signaling and PDGFR have been implicated in the development of Leydig cells and peritubular myoid cells in the testis.^37^, ^38^

Despite a high number of patients receive TKIs like imatinib even beyond oncology, only limited data on their reproductive effects exist.^39^, ^40^, ^41^ This urged us to investigate the impact of imatinib on adult rodent spermatogenic cells and mGSCs ex vivo and in vitro. In this study, we cultured mouse and rat seminiferous tubules to address whether imatinib has an effect on mGSC and spermatogonial proliferation, apoptosis and differentiation in situ. Exposure of mGSCs to imatinib in vitro was utilized to allow targeted long‐term studies of imatinib on stem cells. For comparison, we investigated whether dasatinib had adverse effects similar to imatinib.

## 2 MATERIALS AND METHODS

### 2.1 Imatinib and dasatinib

Imatinib (#ab142070) was purchased from Abcam (Amsterdam, the Netherlands) or received as a generous gift from Novartis (STI571, Glivec). Dasatinib (#ab142050) was ordered from Abcam, the Netherlands. The imatinib doses 1, 3, 5, 10 µM and dasatinib doses 0.01, 0.1, 0.3, 1 µM used in this study were chosen because they are near clinical plasma concentrations detected in patients.^42^, ^43^, ^44^, ^45^, ^46^, ^47^, ^48^ A dose of 100 µM imatinib simulates overdose scenario, however, individuals exposed to this dosage have survived it.^49^, ^50^ Likewise, a dasatinib dose of 10 µM is an overdose, but at least experimental animals have tolerated it without apparent toxic effects.^17^

### 2.2 Animals

Experimental animals (mice and rats) were maintained in the Central Animal Laboratory of University of Turku (Turku, Finland) and B&K Universal AB (Sollentuna, Sweden). All experimental animals were housed under a regular 12 h light/dark cycle in a climate‐controlled room at 21°C ± 3°C with relative humidity of 55% ± 15%. Water and a standard diet (Rat and Mouse breeder and Grower, SDS diets/LBS Biotech, UK) were available ad libitum. Animal husbandry and use were carried out according to Finnish and Swedish laws and following the guidelines of Ethics of Animal Experimentation at University of Turku in accordance with the Guide for Care and Use of Laboratory Animals. The use of experimental animals in this study was approved by University of Turku Ethics Committee for animal experiments. Twenty‐nine rats and 13 mice were used in this study.

### 2.3 Stage‐specific microdissection and culture of rat and mouse seminiferous tubules

Adult (> 2 months old) Sprague–Dawley rats and C57/Bl6J mice were euthanized by cervical dislocation under CO_2_ anesthesia. The testes were dissected and decapsulated. Seminiferous tubule segments representing specific stages of the seminiferous epithelial cycle were cut under a stereomicroscope using the transillumination‐assisted microdissection method, as described previously.^51^, ^52^ The microdissection time was limited to less than 1 h from euthanasia. Four to six seminiferous tubule segments from individual animals were collected for each dose in ex vivo culture experiments. The number of experimental repeats in each tubule culture experiment corresponds to the number of animals used (“*n*”). Staged seminiferous tubule segments of rat and mouse were incubated at 34°C in a humidified atmosphere containing 5% CO₂ for 24, 48, or 72 h in the presence or the absence of imatinib at concentrations of 0, 1, 10, or 100 µM in Dulbecco's Modified Eagle's Medium/Nutrient Mixture F‐12 Ham containing 1× l‐glutamine, 15 mM HEPES, 0.1% bovine serum albumin (BSA) and 100 µg/mL gentamycin. Dasatinib was used at 0, 0.01, 0.1, and 10 µM doses for 24 h or 48 h. DMSO (Sigma‐Aldrich, Saint Louis, USA) was used as a vehicle for dasatinib and therefore added at 1 µL/mL to the control culture medium.

### 2.4 Hoechst staining and flow cytometry analysis of cultured rat seminiferous tubule segments

Twelve 5‐mm long seminiferous tubule segments from stages VII–VIII were dissected from three rats (*n* = 3) and cultured in 0, 1, 10, and 100 µM imatinib and conditions described above for 24 h. The cultured tubule segments were collected by pipetting and washed twice with phosphate‐buffered saline (PBS). Single‐cell suspensions were prepared by enzymatic digestion using Collagenase type IV (Sigma C5138; 1 mg/mL in dulbecco's modified eagle medium) at 37°C for 15 min. Following pelleting at 600×*g* for 5 min at 4°C, and a wash with 2% fetal bovine serum (FBS) in PBS, the cells were resuspended in 500 µL of 2% FBS in PBS and incubated with Hoechst 33342 (1:1000) at room temperature (RT) for 10 min. Finally, the samples were analyzed with NovoCyte Flow Cytometer (ACEA Biosciences Inc.) and FlowJo v10.9 (BD) software.

### 2.5 ^3^H‐thymidine incorporation

Six 2‐mm‐long rat seminiferous tubule segments from stages V, VIIa, and VIII–IX were microdissected and cultured in 0, 1, 10, or 100 µM imatinib, and 100 µM imatinib/100 ng/mL recombinant mouse SCF (Genzyme Transgenics Corp., Cambridge, MA, USA) in conditions described above for 24 h (*n* = 4), 48 h (*n* = 3), or 72 h (*n* = 3). ^3^H‐thymidine (Amersham Pharmacia Biotech, UK, specific radioactivity 5.0 Ci/mmol, final concentration 0.5 µCi) was added to the culture medium 4 h before the end of incubation. The samples were harvested by a Skatron Instruments Cell Harvester (Skatron, Lier, Norway), as previously described.^53^ Briefly, the samples were rinsed with distilled water and collected on glass fiber filter papers (Skatron). Filter discs containing high molecular weight material including DNA, were punched out and transferred to scintillation minivials and Ready Safe scintillation fluid (Beckman Instruments, Fullerton, CA, USA) was added. Incorporated radioactivity was measured by counting in a Beckman LS5000CE scintillation spectrometer as counts per minute (cpm).

### 2.6 Rat seminiferous tubule squash preparations and immunohistochemistry

2‐mm long rat seminiferous tubule segments from stages VIII and IX (*n* = 4) were dissected for culture in 0, 1, 10, and 100 µM imatinib, or 0, 0.01, 0.1, and 10 µM dasatinib, as described above. After a 24‐h culture, seminiferous tubule squash preparations were produced, as previously described.^52^, ^54^ Briefly, the tubule segments were transferred in 20 µL of the culture medium onto a microscope slide and gently squashed between a coverslip and the microscope slide until a cloud‐like cell monolayer was formed. The samples were then snap‐frozen in liquid nitrogen, the coverslip was flipped off using a scalpel, and the sample was first dipped into ice‐cold 96% EtOH and then fixed in 4% PFA for 10 min.

Immunohistochemical staining with apoptosis marker cleaved Caspase‐3 (rabbit ab; #9661S, Cell Signaling, Leiden, the Netherlands) was performed as previously described.^55^ Briefly, the primary antibody was diluted 1:100 in 0.5% BSA in Triton‐X and incubated 1 h at RT in a humid chamber, followed by three washes in PBS. The secondary antibody (Envision + System‐HRP‐labeled polymer anti‐rabbit ab (K4003, Dako North America, CA, USA; undiluted) was applied for 30 min. Following three washes in PBS, diaminobenzidine (DAB) (Dako North America, CA, USA) was added for 2 min. Counter‐staining was done with hematoxylin and eosin, and mounting with Aquatex (Merck, Germany). The number of cleaved Caspase‐3‐positive cells was manually quantified, and reported as number of positive cells per 1 mm of seminiferous tubule.

### 2.7 Isolation and culture of mouse embryonic fibroblasts

Pregnant mouse dams of mixed genetic background were sacrificed by CO_2_ asphyxiation and cervical dislocation at gestational day 13.5, counting the day of the plug as 0.5. The uterus was dissected and moved to a petri dish containing PBS. The embryos were released by making an incision to the uterine horn. Heads of embryos were cut off and internal organs were removed under a stereomicroscope. Remaining tissues were transferred to 6‐well plates (one embryo per well), cut into small pieces and enzymatically digested using trypsin (0.6 mg/mL; Worthington) and DNase I (4.5 U/mL; Worthington) in total 15 min at 37°C and dissociated by pipetting. The cell suspensions were mixed with culture medium and transferred to petri dishes for culture. When cultures were confluent, MEFs were harvested using trypsin/ethylene diamine tetraacetic acid (EDTA). MEFs from passages 3−15 were used as feeders for mGSCs.

### 2.8 Isolation and culture of male germline stem cells

Four adult (> 8 weeks old) male mice from DBA/2JRccHsd strain (Envigo, Venray, the Netherlands) were sacrificed, and the testes were dissected and decapsulated. mGSCs were isolated and cultured, as previously described.^56^ Briefly, single‐cell suspensions were prepared by enzymatic digestion: collagenase type I (0.1 mg/mL; Worthington) 10 min at 37°C, followed by trypsin (0.6 mg/mL; Worthington) and DNase I (4.5 U/mL; Worthington) 5 min at 37°C. Seminiferous tubule fragments were dissociated by pipetting in 10% FBS in PBS and passed through a 70‐µm cell strainer. Following centrifugation at 600×*g*, 5 min, the cell pellets were resuspended in 2% FBS in PBS and spermatogonia (including mGSCs) were isolated by biotinylated CD9 antibody (Biolegend clone MZ3, 1:500) selection using an EasySep biotin selection kit (Stem Cell Technologies) according to manufacturer's instructions. Finally, the CD9‐selected cells were resuspended in the mGSC culture medium consisting of StemPro‐34 media (Thermo Fischer Scientific) supplemented with 5 mg/mL BSA (Sigma‐Aldrich, A9418), 6 mg/mL Glucose, l‐Glutamine (1×, Gibco), Pen/Strep (1×), MEM Vitamin solution (1×, Gibco), MEM Nonessential amino acid solution (1×, Gibco), 0.5 µM 2‐mercaptoethanol (Gibco), 60 µM Putrescine, 0.1 mM ascorbic acid (Stem Cell Technologies), 340 µµ Pyruvate (Sigma), 1 µL/mL lactic acid (Sigma), 10 µg/mL Biotin (Sigma‐Aldrich), 25 µg/mL insulin (Sigma), 100 µg/mL Transferrin (Sigma‐Aldrich), 30 nM sodium selenite, 30 ng/mL beta‐estradiol (Sigma), 60 ng/mL Progesterone (Sigma), 1% FBS (Gibco), StemPro‐34 supplement (1×), 20 ng/mL human EGF (Gibco), 10 ng/mL murine bFGF (Peprotech) and 10 ng/mL human glial cell line‐derived neurotrophic factor (GDNF) (Peprotech) and plated on mitomycin‐inactivated (10 µM, 4 h) MEFs.

mGSC cultures were maintained at 37°C, 5% CO_2_, and passaged every 3−5 weeks using trypsin/EDTA. Passage 4−6 mGSCs from two independent lines were used for experimentation. For proliferation analysis, mGSCs were seeded in 24‐well plates at low density and incubated in 0, 1, 3, 5, and 10 µM imatinib or 0, 0.01, 0.1, 0.3, and 1 µM dasatinib for 5 days, 7 days, or 4 weeks. Control conditions in dasatinib experiments included 1 µL of DMSO/1 mL of the tubule culture medium. The cell number was quantified using TC20 automated cell counter (Bio‐Rad). Trypan Blue was used to detect dead cells in the samples. For apoptosis, Annexin V‐FITC Early Apoptosis Detection Kit (Cell Signaling, #6592) was used according to the manufacturer's instructions. For differentiation analysis, mGSCs were subcultured until they formed grape‐like colonies, followed by exposure to 0, 1, 5, and 10 µM imatinib for 3 days. Retinoic acid (RA, 1 µM; Sigma‐Aldrich R2625) was applied for the last 48 h. Cells were treated with trypsin/EDTA, reconstituted in 2% FBS in PBS and stained with c‐KIT‐APC (1:200; eBioscience, 17‐1171‐82) and MCAM‐FITC antibodies (1:200; BioLegend, 134708). DAPI (1:10 000) was used for live/dead discrimination. The samples were analyzed using NovoCyte flow cytometer (ACEA) and FlowJo v10.9 software.

### 2.9 Induction of spermatogonial differentiation ex vivo

Mouse seminiferous tubule segments from stages II–VI were dissected for an ex vivo differentiation induction experiment. The tubule segments were first incubated 2 h in 0, 1, and 10 µM imatinib. RA (1 µM) was then added for 22 h. At the end of culture, the tubule segments were collected by pipetting and washed twice in ice‐cold PBS. The samples were prepared for whole‐mount immunofluorescence staining using STRA8 (1:500; Abcam, ab49405), c‐KIT (1:300; Invitrogen, 17‐1171‐82) and LIN28A (1:500; R&DSystems, AF3757) antibodies as described in 2.10, or for flow cytometry using c‐KIT antibody (1:100, eBioscience, 17‐1171‐82). For quantitation of LIN28A‐positive/STRA8‐positive or negative cells, 40 randomly chosen areas were imaged (3i CSU‐W1 spinning disk confocal microscope; 200× magnification; 3i Intelligent Imaging Innovations) and systematically quantified. On average 644 (± 153) LIN28A‐positive cells were quantified for STRA8‐positivity/negativity per sample (*n* = 2 per group). In flow cytometry analysis, c‐KIT positive cells were divided into c‐KIT+ and c‐KIT‐high subsets (*n* = 3 per group).

### 2.10 Male germline stem cell proliferation ex vivo and mouse seminiferous tubule whole‐mount immunostaining

Mouse seminiferous tubule segments from stages IX–I were dissected for a 48‐h culture in 0 and 10 µM imatinib and dasatinib. Control conditions in dasatinib experiments included 1 µL of DMSO/mL of the tubule culture medium. Four and three adult male mice from C57Bl/6J background were used for imatinib (*n* = 4) and dasatinib (*n* = 3) experiments, respectively. Following culture, the tubule segments were washed three times in ice‐cold PBS, fixed and stained, as previously described.^52^, ^57^ Briefly, the tubules were fixed (4% PFA) 6 h at +4°C on a rotating table, followed by two washes with PBS. Following 1‐h blocking in 2% BSA + 10% FBS in 0.3% Triton X‐100 in PBS, the samples were stained overnight at +4°C on a rotating table with primary antibodies: GFRα1 (1:250; RnD systems, AF560) and KI67 (1:200; Invitrogen, 14‐5698‐82). Following three washes in PBS, 2‐h incubation at RT on a rotating table with respective secondary antibodies (1:500; Invitrogen, A11056, A21208) was done. Finally, after three washes in PBS, the tubules were poured and mounted onto a microscope slide. Forty randomly chosen areas were selected for 3i CSU‐W1 spinning disk confocal microscopy imaging (3i) and systematically quantified for GFRα1‐positive/KI67‐positive or negative cells. On average 766 (± 118) GFRα1‐positive cells were quantified for KI67‐positivity/negativity per sample.

### 2.11 Statistical methods

All data were analyzed for statistically significant differences using one‐way ANOVA, *t*‐test, or non‐parametric test (Kruskal–Wallis), followed by Tukey's or Dunn's multiple comparison test on GraphPad Prism 5 (GraphPad Software, Inc., La Jolla, CA, USA). The significance level was at *p* < 0.05.

## 3 RESULTS

### 3.1 Imatinib reduces the number of DNA‐synthesizing cells in cultured rat seminiferous tubules

**FIGURE 1:**
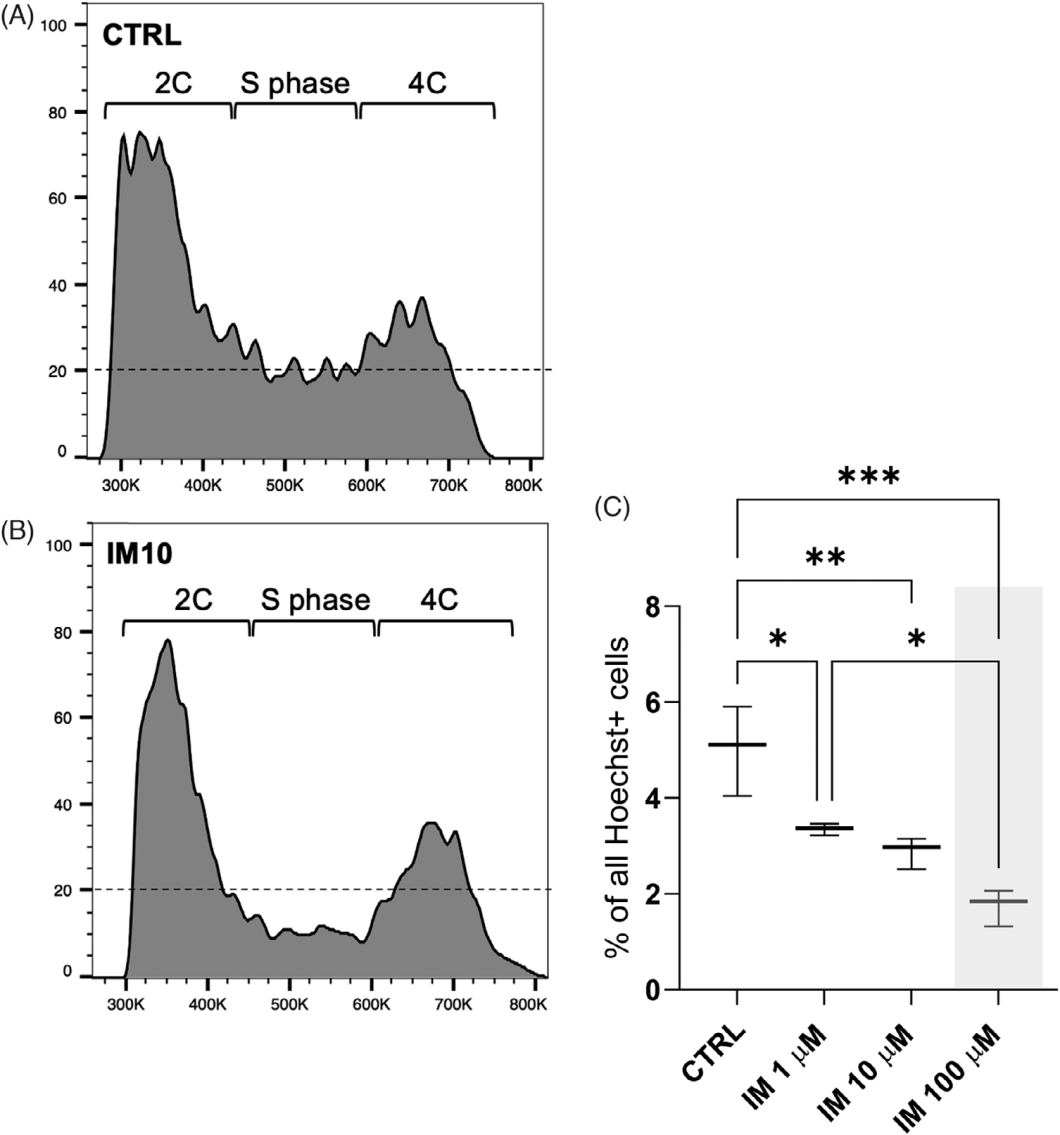
**Imatinib decreases the percentage of cells undergoing DNA synthesis in cultured rat seminiferous tubules**. Seminiferous tubule segments from stages VII–VIII were cultured for 24 h in the absence or presence of imatinib (IM) (1, 10, and 100 µM). Single‐cell suspensions were prepared and stained with chromatin dye Hoechst 33342, and the distribution of cell populations was analyzed by flow cytometry (gating is shown in Figure S1). Representative histograms of 2C, S phase and 4C populations from (A) CTRL and (B) IM10 samples are shown. (C) The percentage of cells in the S phase of the cell cycle decreased in the presence of imatinib in a dose‐dependent fashion. Acute toxic dose of IM (100 µM) is highlighted with a grey box. One‐way ANOVA, followed by Tukey's multiple comparison test. Median, ± max and min, *n* = 3. ***p < 0.001, **p < 0.01, *p < 0.05.

Despite the fact that imatinib inhibits a number of tyrosine kinases essential for testicular development and physiology, very little is known about its effect on spermatogenic cells in the adult testis. To this end, we dissected rat seminiferous tubule segments from epithelial stages VII–VIII for culture in 0, 1, 10, and 100 µM imatinib. These stages were selected because they are relatively easy to dissect in large quantities, and within the time window of a 24‐h culture many key spermatogenic events are expected to take place, including spermatogonial differentiation followed by S phase, preleptotene‐to‐leptotene transition and meiotic DNA synthesis, onset of spermatid elongation, and spermiation. Single‐cell suspensions were prepared from the cultured tubule segments and stained with Hoechst 33342 chromatin dye, that also penetrates living cells, and analyzed with flow cytometry. We were able to clearly discriminate 1C (spermatids), 2C (consisting of somatic cells and spermatogonia, preleptotene spermatocytes), and 4C (leptotene and pachytene spermatocytes) populations (Figure S1) in all samples. Consistent changes between control and imatinib‐treated samples were observed only in the number of cells in the S‐phase of the cell cycle (Figure 1A,B) that dose‐dependently decreased in the presence of imatinib (Figure 1C). These data suggest that DNA‐synthesizing cells (spermatogonia and early spermatocytes) are affected by short‐term imatinib exposure ex vivo.

### 3.2 Imatinib reduces ^3^H‐thymidine incorporation in cultured rat seminiferous tubules

**FIGURE 2:**
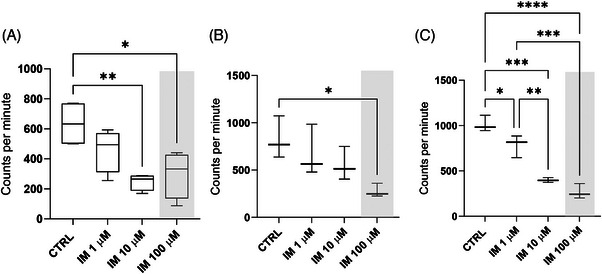
**Imatinib decreases ^3^H‐thymidine incorporation in cultured rat seminiferous tubules**. Seminiferous tubule segments from stages (A) VIII–IX (24 h), (B) V (72 h), and (C) VIIa (72 h) were cultured in the absence or presence of imatinib (1, 10, and 100 µM) to record ^3^H‐thymidine incorporation. Imatinib decreased both meiotic (A), (C) and mitotic (B) ^3^H‐thymidine incorporation. Acute toxic dose of imatinib (100 µM) is highlighted with a grey box. One‐way ANOVA, followed by Tukey's multiple comparison test. Median, ± max and min, *n* = 4 (24 h exposure), *n* = 3 (72 h exposure). ****p < 0.0001, ** *p* < 0.01, *p < 0.05.

The decrease in the number of cells in the S phase can be explained by two things: either DNA synthesis in spermatogonia or early spermatocytes is disrupted and/or delayed due to exposure to imatinib, or imatinib induces apoptosis of these cells. To this end, we first dissected rat seminiferous tubule segments from stages I, V, VIIa, and VIII–IX for culture in imatinib at the conditions mentioned above in the presence of ^3^H‐thymidine. After 24 and 72 h culture, we were able to record a dose‐responsive decline in the amount of ^3^H‐thymidine incorporation, corroborating the flow cytometry finding about diminished number of DNA‐synthesizing cells in the presence of imatinib in stages VIII–IX at 24 h and VIIa at 72 h (Figure 2A–C). These data suggest that imatinib impinges on both meiotic DNA synthesis^58^, ^59^ (in preleptotene spermatocytes; 24‐h culture of stages VIII‐IX and 72‐h culture of stages VIIa, respectively; Figure 2A,C) and mitotic DNA synthesis (in type B spermatogonia; 72‐h culture of stage V; Figure 2B).

### 3.3 Imatinib induces apoptosis dose‐dependently in cultured rat seminiferous tubules

**FIGURE 3:**
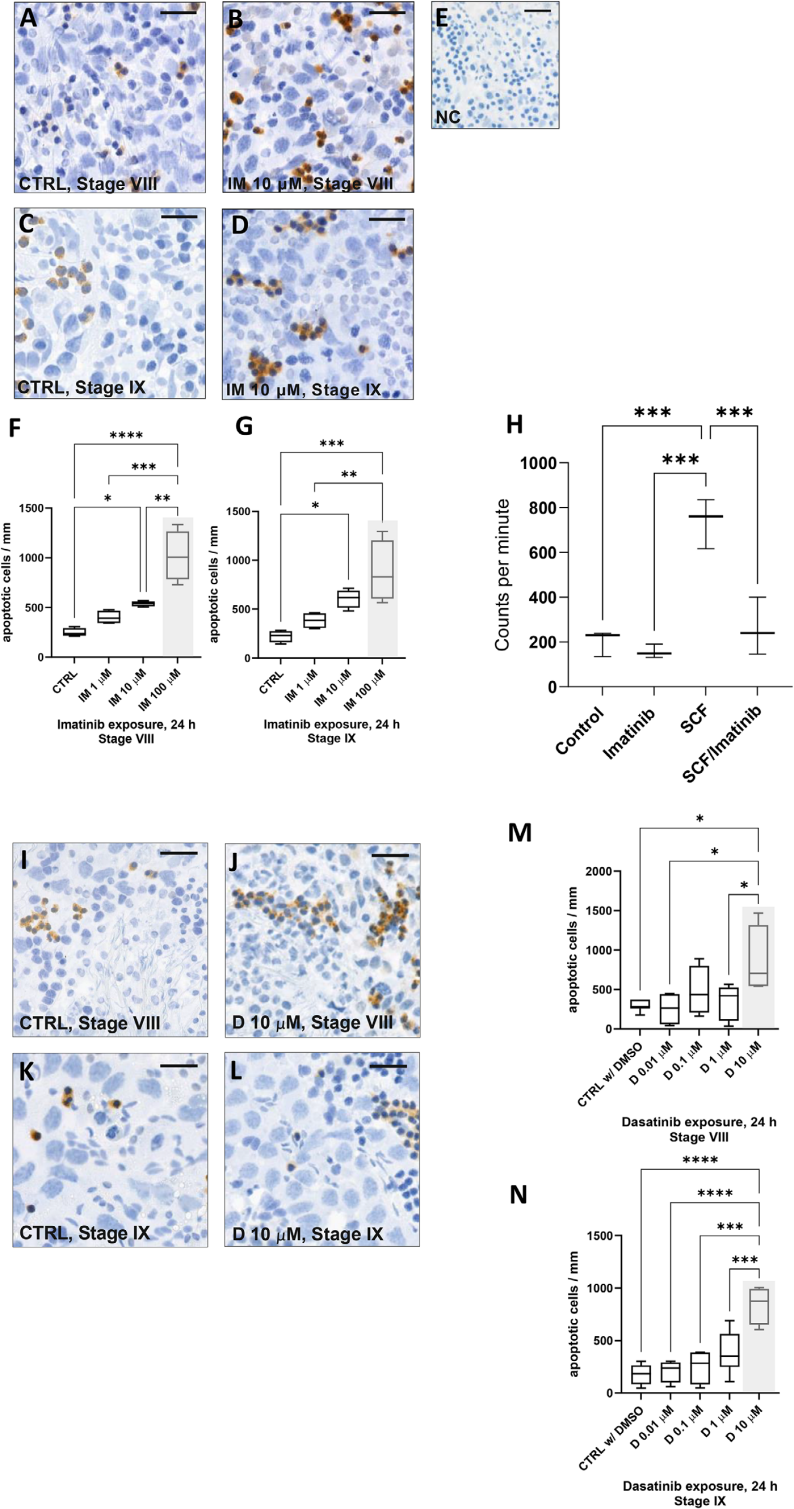
**Imatinib, but not dasatinib, induces apoptosis in cultured rat seminiferous tubule segments with clinically relevant doses**. Seminiferous tubule segments were cultured for 24 h in the absence or presence of imatinib (1, 10, and 100 µM) followed by preparation of monolayers (squash) and staining of cleaved Caspase‐3 (DAB chromogen). Cleaved Caspase‐3‐positive cells were observed in all samples. Representative stainings from (A) stage VIII CTRL, (B) stage VIII imatinib 10 µM, (C) stage IX CTRL, and (D) stage IX imatinib 10 µM are shown. (E) Negative control staining. Scalebars 50 µm. (F) Quantitation of cleaved Caspase‐3‐positive cells in imatinib‐exposed tubule segments from stage VIII and (G) stage IX. A dose‐dependent increase was observed in imatinib‐exposed tubule segments (F,G). (H) SCF (100 ng/mL) increased ^3^H‐thymidine incorporation in cultured rat stage XII seminiferous tubule segments. The effect of SCF was abolished by imatinib. As a comparison seminiferous tubule segments were also cultured in the absence or presence of dasatinib (D) 0.01, 0.1, 1 and 10 µM. (I) Representative stainings of stage VIII CTRL w/ DMSO, (J) stage VIII dasatinib 1 µM, (K) stage IX CTRL w/ DMSO and (L) stage IX dasatinib 1 µM are shown. Scalebars 50 µm. (M) Quantitation of cleaved Caspase‐3‐positive cells in dasatinib exposed stage VIII and (N) stage IX. Acute toxic doses of IM (100 µM) and D (10 µM) are highlighted with a grey box. One‐way ANOVA, followed by Tukey's multiple comparison test. Median, ± max and min, *n* = 4 in cleaved Caspase‐3 quantifications (whereas *n* = 8 in dasatinib 1 µM and stage IX CTRL w/ DMSO, *n* = 7 in stage VIII CTRL w/ DMSO), *n* = 3 in ^3^H‐thymidine incorporation. ****p < 0.0001, ***p < 0.001, **p < 0.01, *p < 0.05. SCF, Stem cell factor.

We then investigated whether imatinib‐induced apoptosis might explain the cellular changes in cultured rat seminiferous tubules. Segments from stages VIII and IX were cultured in 0, 1, 10, and 100 µM imatinib for 24 h, processed to squash preparations and stained for cleaved Caspase‐3, an early apoptosis marker.^60^ (Figure 3A–E). Quantitative analysis showed that clinically relevant doses of imatinib dose‐dependently increased the number of cleaved Caspase‐3‐positive cells demonstrating that, similar to cancer cells,^61^, ^62^ imatinib induces apoptosis in spermatogenic cells (Figure 3F,G).

### 3.4 Imatinib blocks the effect of stem cell factor in cultured rat seminiferous tubules

Given the fact that induction of apoptosis in imatinib‐treated seminiferous tubules (Figure 3F,G) could also explain the reduction in DNA synthesis (Figure 2), we wanted to further investigate the effect of imatinib on blocking the function of SCF, a critical survival factor for germ cells.^33^, ^34^, ^63^ To this end, we dissected rat seminiferous tubule segments from stage XII for culture with imatinib and SCF. Stage XII was selected because we have previously shown that SCF promotes survival of DNA synthesizing spermatogonia in that stage.^33^ After a 24‐h culture, we measured a small but significant decrease in DNA synthesis caused by imatinib alone, corroborating the earlier data (Figure 2A–C). Notably, SCF administered at 100 ng/mL triggered a higher than three‐fold increase in ^3^H‐thymidine incorporation, which was abolished by imatinib (Figure 3 H). Together these data suggest that the primary effects of imatinib on differentiating male germ cells (type A1 to B spermatogonia and preleptotene spermatocytes) are mediated through inhibition of SCF/c‐KIT signaling, resulting in increased cell death and perturbed DNA synthesis, thus functionally impairing spermatogenesis.

### 3.5 Dasatinib does not induce apoptosis in rat seminiferous tubules after 24‐h culture with clinically relevant doses

In differentiating germ cells, dasatinib may also disturb the SCF/c‐KIT signaling, since dasatinib also inhibits c‐KIT.^16^ Therefore, we wanted to investigate whether dasatinib induces apoptosis in rat seminiferous tubule segments at stages VIII and IX. Tubule segments from those stages were cultured for 24 h with or without dasatinib (0, 0.01, 0.1, and 10 µM), then squashed and stained for cleaved Caspase‐3 (Figure 3I–L). Only the highest dose (10 µM), but not the clinically relevant doses (0.01–1 µM) of dasatinib, induced apoptosis in rat seminiferous tubules at stages VIII and IX (Figure 3M,N).

### 3.6 Imatinib and dasatinib decrease male germline stem cell colony growth in vitro

Given the fact that applicability of seminiferous tubule culture for the toxicological study of spermatogenesis is limited to short‐term cultures,^54^ we moved to study imatinib's effects also on mGSCs that can be propagated virtually indefinitely in vitro.^64^ Moreover, while the effects on spermatogenic cells are transient and relieved when the stressor is removed, the effects on mGSCs are more permanent because they are the foundation of lifelong spermatogenesis and present in the testis across the lifespan of an individual. We also moved to use the mouse as a model organism because mGSC culture has been established better in mouse than any other mammalian enabling unlimited propagation of mGSCs ex vivo. We established mouse mGSC cultures on mitomycin‐inactivated mouse embryonic fibroblasts (MEFs) by selecting CD9‐positive cells from adult mouse testes using MACS, as previously described.^56^ mGSCs on culture were validated by characteristic grape‐like colony morphology^64^ and melanocyte cell adhesion molecule^65^ (MCAM) and SALL4 (Spalt‐like 4^66^) expression (Figure S3).

**FIGURE 4:**
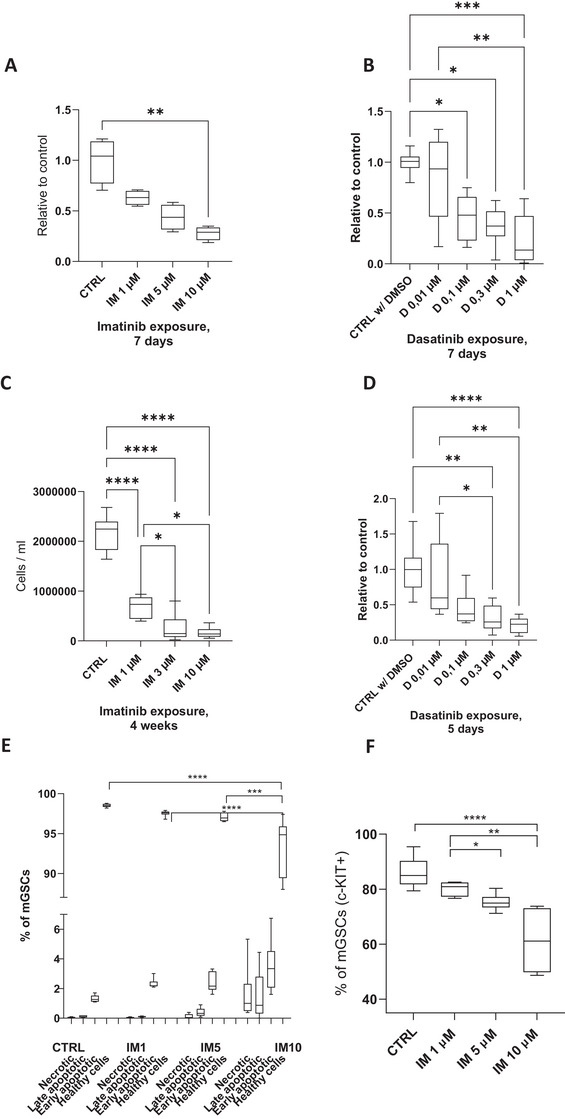
**Imatinib and dasatinib decrease mGSC colony growth in vitro**. (A) Imatinib decreased mGSC colony growth after 7 days of culture, *n* = 12. (B) Dasatinib also decreased mGSC colony growth after 7 days of culture, *n* = 9. (C) Imatinib decreased mGSC propagation after 4 weeks of culture, *n* = 6. (D) mGSCs which were directly exposed to dasatinib when plated, grew significantly less after 5 days exposure, *n* = 10. (E) Imatinib did not induce apoptosis in cultured mGSCs, but the number of healthy cells was decreased in IM10, *n* = 6. (F) Imatinib dose‐dependently decreased the number of c‐KIT‐expressing cells after a 48‐h treatment of mGSCs with 1 µM retinoic acid (RA), *n* = 6. The Kruskal–Wallis test (non‐parametric) followed by Dunn´s multiple comparisons test (A) and one‐way ANOVA, followed by Tukey's multiple comparison test (B–D). Median, ± max and min. ****p < 0.0001, ***p < 0.001, **p < 0.01, *p < 0.05. mGSCs, Male germline stem cells.

We wanted to evaluate the effects of imatinib on mGSC proliferation, apoptosis, and differentiation capacity. Dasatinib was used as another TKI in the mGSC experiments. First, the outcome of imatinib and dasatinib exposure on mGSC propagation was studied using two experimental setups. The dosages of imatinib and dasatinib in these experiments were determined based on meticulous investigation of the effects on MEF feeders allowing the direct effects of imatinib and dasatinib on mGSCs to be studied (Figures S4A–O and S5A–M). Doses of 1−10 µM imatinib and 0.01–1 µM dasatinib were chosen because these dose ranges did not affect significantly the viability, cell growth or morphology of MEFs (Figures S4I,J,O and S5K–M). We then passaged mGSCs on 24‐well plates at a standard cell density and cultured them for 7−15 days until they formed small colonies (Figures S6A,B and S7A–D). Imatinib (0, 1, 5, and 10 µM) or dasatinib (0, 0.01, 0.1, 0.3, and 1 µM) was then added for 7 days, followed by scoring of the cell number. Trypan Blue was used to detect cell death beside the cell calculation. Interestingly, there was a dose‐dependent decline in the number mGSCs (Figure 4A,B) showing that both imatinib and dasatinib adversely affect mGSC colony growth.

Because cell density in mGSC cultures affects the composition of mGSC populations by promoting progenitor‐like characteristics at higher densities^56^, we investigated the effects of imatinib and dasatinib treatment prior to mGSC colony formation. Consequently, imatinib and dasatinib exposure was initiated during passaging at the stage when mGSC are plated as single cells on feeder layers. The cultures were established on 24‐wells at a low cell density (3–4 thousand mGSCs per well) and maintained with imatinib for 4 weeks, corresponding to the standard subculturing interval for mGSCs in our laboratory. While the control wells reached high confluence, the number and size of mGSC colonies in imatinib‐exposed wells was clearly reduced (Figure 6E–L). This was confirmed by cell counting showing 70%−90% dose‐dependent decline in mGSC number in the presence of imatinib (Figure 4C) providing further evidence that mGSC propagation and/or survival are compromised in the presence of imatinib. With dasatinib, a pronounced reduction in cell growth was evident already after 5 days, which led us to prematurely terminate the experiment (Figure 4D and Figure S7I–P).

The number of mGSCs in culture is defined by their proliferation rate and occurrence of cell death. To evaluate the possible cell death and apoptosis more in detail, we studied cell viability of mGSCs cultured with or without imatinib (0, 1, 5, and 10 µM) using Annexin V cell death analysis flow cytometry kit. Based on staining for Annexin V (AV; early apoptosis marker) and propidium iodide (PI; for dead cell discrimination) the cultured mGSCs were divided into healthy (AV‐neg/PI‐neg), early apoptotic (AV‐pos/PI‐neg), late apoptotic (AV‐pos/PI‐pos), and necrotic cells (AV‐neg/PI‐pos). The proportion of healthy cells remained at 95%−98% in all studied conditions (Figure 4 E), although the reduction in the percentage of healthy cells in 10 µM imatinib was significantly higher than in 0, 1, and 5 µM imatinib.

### 3.7 Imatinib impinges on male germline stem cell differentiation in vitro

mGSCs on culture readily respond to retinoic acid (RA) and start to express markers of differentiating spermatogonia, such as c‐KIT and stimulated by retinoic acid gene 8 (STRA8), emulating transition of undifferentiated spermatogonia to type A1 differentiating spermatogonia at epithelial stages VII–VIII in vivo.^56^ *^,^* ^67^ To address whether imatinib perturbs this decisive cell fate shift, we passaged mGSCs at a standard density on 24‐wells and allowed them to grow for 2 weeks. We then treated the cultures with increasing doses of imatinib (0, 1, 5, and 10 µM) and 1 µM RA. The samples were then processed to single‐cell suspensions and analyzed by flow cytometry for c‐KIT expression (Figure S8A,B). While in control cultures around 83% of spermatogonia were found c‐KIT‐positive, a dose‐dependent reduction was seen in the percentage of c‐KIT‐positive spermatogonia in the presence of imatinib (Figure 4F). These data suggest that imatinib inhibits mGSC differentiation in vitro.

### 3.8 Imatinib does not block A_undiff_ differentiation but suppresses c‐KIT expression in nascent differentiating spermatogonia

**FIGURE 5:**
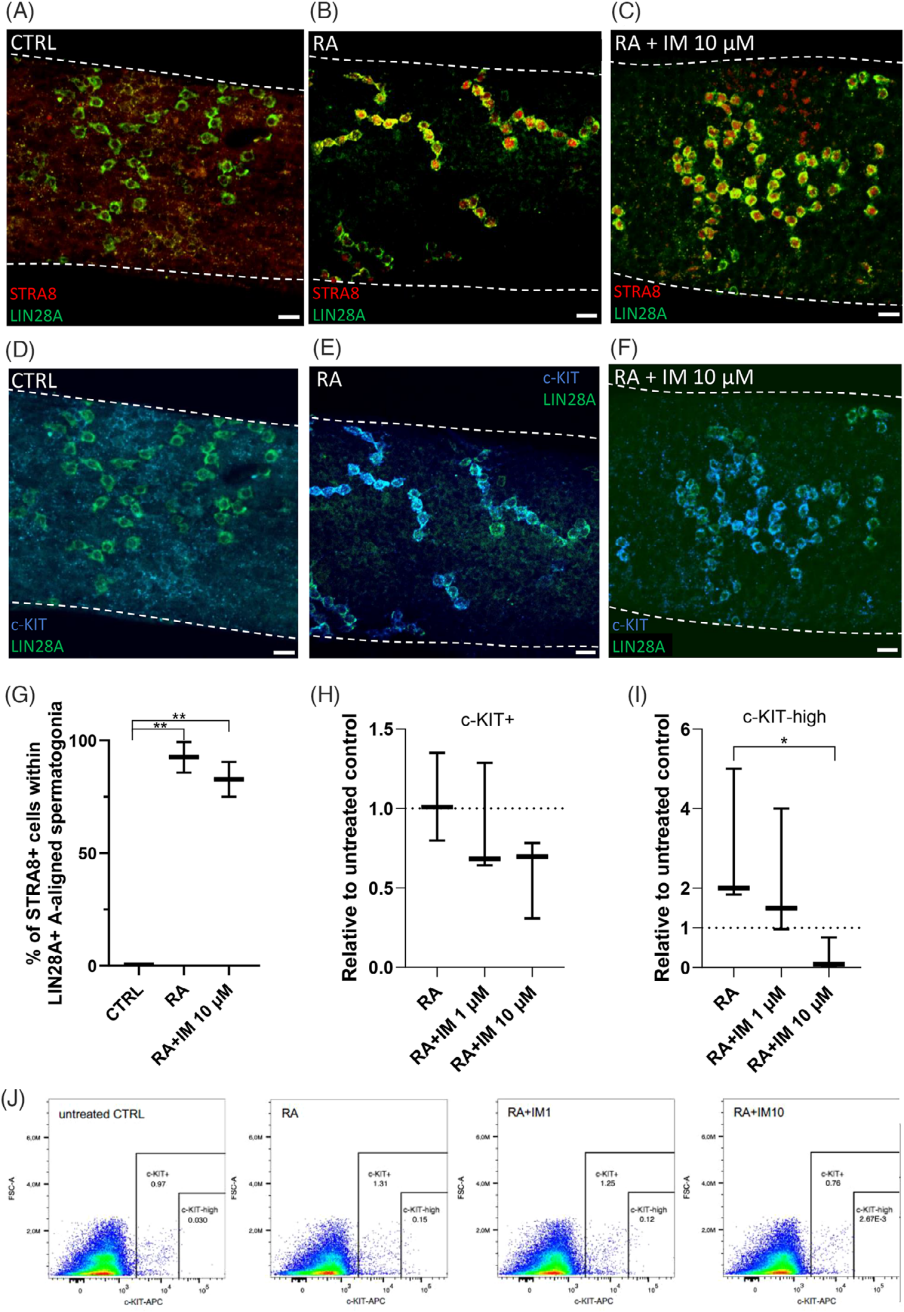
**Imatinib does not interfere with mGSC differentiation induction ex vivo, but reduces c‐KIT expression in nascent differentiating spermatogonia**. (A) Stages II–VI mouse seminiferous tubule segments were cultured for 24 h in control conditions, (B) 1 µM retinoic acid (RA), and (C) 1 µM RA plus imatinib 10 µM and stained for LIN28A and STRA8. Scalebars 10 µm. (D–F) a co‐staining for c‐KIT confirms the RA induced expression of both STRA8 (A–C) and c‐KIT (D–F) in LIN28a+ A_undiff._ (G) Percentage of STRA8+ positive spermatogonia within the LIN28A+ A‐aligned spermatogonial subset. (H) Flow cytometry analysis of c‐KIT expression after RA‐induced differentiation showing the proportion of c‐KIT‐positive cells (relative to untreated control; *n*= 1), and (I) the proportion of c‐KIT‐high nascent spermatogonia (relative to untreated control; *n*= 1). (J) Dot‐plots of c‐KIT flow cytometry results showing the c‐KIT‐positive and c‐KIT‐high populations in CTRL, RA‐treated, and RA+IM10‐treated samples. *n* = 3. One‐way ANOVA, followed by Tukey´s multiple comparison test (G) or Kruskal–Wallis test (non‐parametric) followed by Dunn´s multiple comparisons test (H and I). Median, ± max and min. **p < 0.01, *p < 0.05. mGSCs, Male germline stem cells.

We then investigated whether imatinib can dampen the differentiation‐inducing effect of RA in cultured seminiferous tubules. For these experiments, we dissected segments from stages II–VI. These stages were selected because previous studies have shown that progenitor A_undiff_ spermatogonia become sensitive to RA between stages II–VI.^68^, ^69^ Following a 24‐h culture in the presence or the absence of imatinib (0, 1, and 10 µM) and 1 µM RA, the tubule segments were fixed and stained with LIN28A antibody, which displays enriched expression in 4–16‐cell syncytia of A_undiff_ (A‐aligned_4‐16_) spermatogonia primed for differentiation^70^, ^71^, and differentiation markers STRA8 and c‐KIT (Figure 5A–F). Reassuringly, untreated stages II–VI tubule segments that were not exposed to RA were devoid of STRA8 and c‐KIT staining, whereas in RA‐exposed tubules vast majority of LIN28A‐positive A‐aligned_4‐16_ spermatogonia was also found positive for STRA8 and c‐KIT irrespective of the presence or absence of imatinib (Figure 5B,C E–G), suggesting that imatinib does not interfere with A_undiff_ spermatogonia differentiation induction ex vivo.

While this discrepancy between in vitro and ex vivo findings might be attributed to differences in the experimental setting, we went on to study whether the expression levels of c‐KIT, the molecular target of imatinib, are affected by it. To this end, we used the same experimental setup, but instead of whole‐mount immunohistochemistry, we prepared cell suspensions for c‐KIT staining and flow cytometry. In all studied conditions (with or without RA and/or imatinib), an even subset of cells was positive for c‐KIT (c‐KIT+), representing primarily intermediate and type B spermatogonia (Figure 5H). Notably, in response to RA, a subset of stage II–VI cells was found to express high levels of c‐KIT (designated c‐KIT‐high; around 0.2% of all cells; Figure 5J), likely representing the nascent A1 spermatogonia—a population formed in response to RA. Interestingly, the c‐KIT‐high population was absent in RA + 10 µM imatinib‐treated samples (Figure 5I,J). In summary, these data suggest that while imatinib does not inhibit induction of spermatogonial differentiation ex vivo per se (STRA8 is induced normally), it downregulates c‐KIT expression in differentiating spermatogonia (they fail to express high levels of c‐KIT), providing a plausible mechanism for its proapoptotic effect, given the prosurvival role of SCF/c‐KIT signaling in spermatogenic cells.^33^, ^34^, ^63^

### 3.9 Imatinib, but not dasatinib, reduces the number of KI67‐positive A_undiff_ spermatogonia ex vivo

**FIGURE 6:**
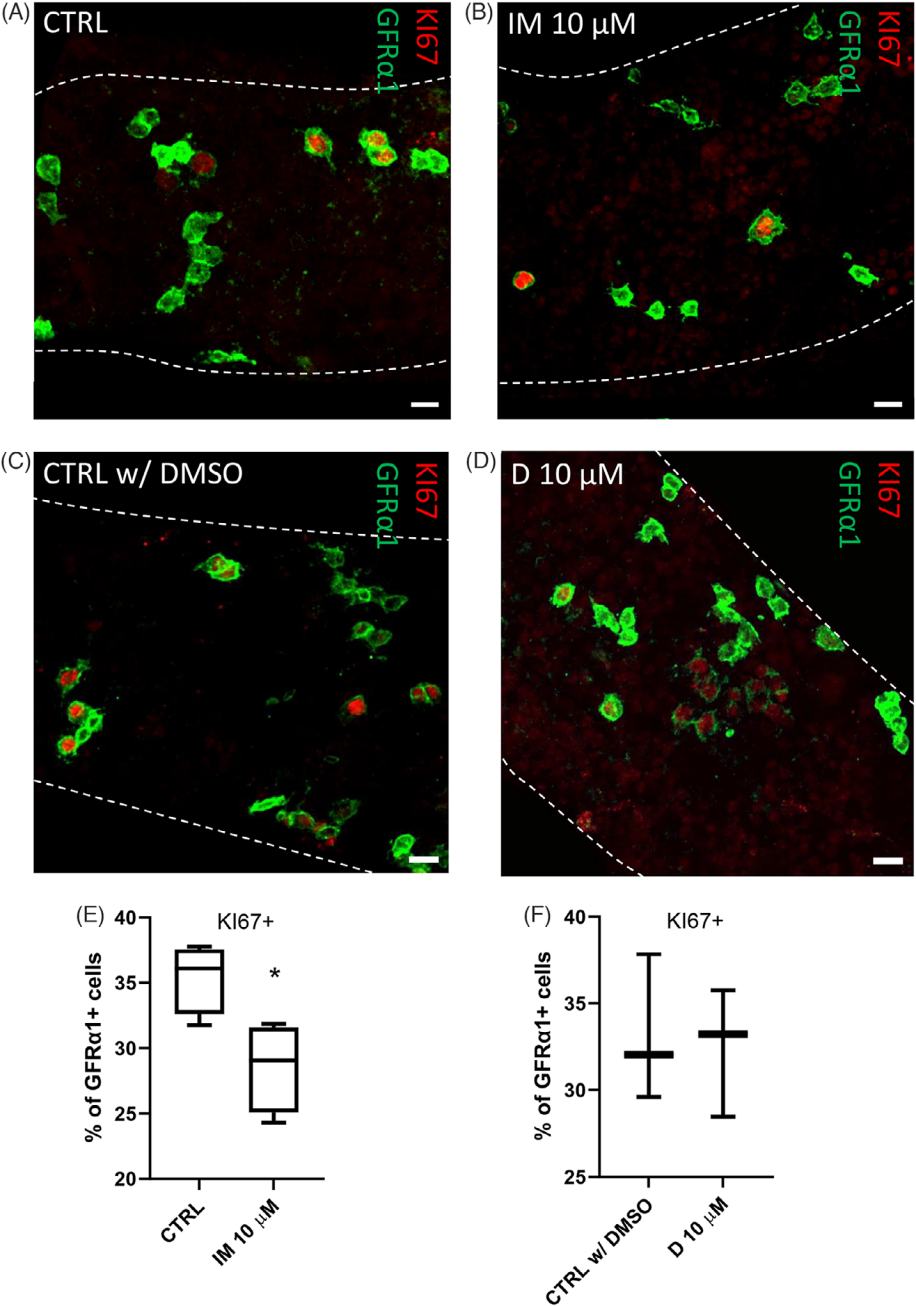
**Imatinib, but not dasatinib, reduces the number of KI67‐positive mGSCs ex vivo**. (A and C) Stages IX–I mouse seminiferous tubule segments were cultured 48 h in the absence (CTRL or CTRL w/ DMSO) or (B) presence of 10 µM imatinib or (D) dasatinib and stained for GFRα1 and KI67. Scalebars 10 µm. (E) The percentage of GFRα1‐positive cells found to express KI67 was reduced in the presence of imatinib, (F) but not in the presence of dasatinib. *T*‐test, median, ± max and min, *n* = 4 (imatinib), *n* = 3 (dasatinib). *p < 0.05. mGSCs, Male germline stem cells.

A_undiff_ spermatogonia are the in vivo counterparts of cultured mGSCs.^56^ Given the fact that in vitro culture of mGSCs does not fully recapitulate the stem cell niche in the testis, we wanted to investigate the effects of imatinib and dasatinib on A_undiff_ proliferation and differentiation in situ using ex vivo mouse seminiferous tubule culture. We first dissected mouse seminiferous tubule segments from stages IX–I for a 48‐h culture in 0 and 10 µM imatinib and dasatinib. These stages were selected because A_undiff_ are considered to self‐renew in stages X–II.^72^, ^73^ Following culture, the tubule segments were fixed and stained with GDNF family receptor alpha 1 (GFRα1), a specific marker of the self‐renewing A_undiff_; and KI67, a proliferation marker (Figure 6A–D). In control samples around 37% of GFRα1‐positive cells was found to be KI67‐positive, whereas in the presence of 10 µM imatinib, the proportion fell to 29% (Figure 6E), corroborating our in vitro finding concerning the anti‐proliferatory effect of imatinib on stem and progenitor cells of the male germline. In contrast, in dasatinib‐exposed tubule segments there was no significant difference in the percentages of KI67‐positive GFRα1‐positive cells as compared to control (32% in CTRL vs. 33% in dasatinib 10 µM) (Figure 6F).

## 4 DISCUSSION

Imatinib is a first‐generation TKI which made a clinical breakthrough in the treatment of CML and GIST as a target‐specific cancer drug.^2^ Subsequently, the use of imatinib has spread not only to other fields of oncology, but also to inflammatory diseases proving its immunomodulatory functions.^74^ Most recently imatinib has been suggested as a potential therapeutic agent for pediatric patients with type 1 diabetes.^13^ Before imatinib is introduced as a treatment for new indications, the side effect profile needs to be well investigated, including possible adverse effects on fertility. Therefore, in this study we used adult rodent models to study the effects of imatinib on spermatogenic cells and mGSCs, using ex vivo seminiferous tubule culture and mGSC propagation in vitro. After observing the adverse effects of imatinib, we aimed to assess whether dasatinib, a second‐generation TKI, exhibits a comparable reproductive toxicity profile.

One of the key findings of our study is the dose‐dependent reduction in mGSC proliferation in vitro. After 4 weeks of culture, the number of mGSCs was 70%−90% lower in the presence of imatinib (1–10 µM). In dasatinib‐exposed mGSCs, we also observed a dose‐dependent decline in cell growth. We further corroborated those findings for imatinib using an ex vivo seminiferous tubule culture setting. Segments representing mouse epithelial stages IX–I were dissected for that because A_undiff_ spermatogonia have been shown to exhibit proliferative activity in stages X–II when self‐renewal factor GDNF is also found at a high level.^72^, ^73^ Importantly, about one‐third of GFRα1‐positive A_undiff_ cultured in their niche in control conditions were found positive for KI67. This is comparable or slightly higher than previously reported for GFRα1‐expressing cells in vivo^57^ showing that stage‐dependent events in the A_undiff_ niche can be recapitulated in short‐term ex vivo seminiferous tubule culture. Notably, the fraction of KI67‐positive GFRα1‐expressing A_undiff_ fell to 29% in the presence of 10 µM imatinib, whereas non‐significant difference was found in dasatanib‐exposed seminiferous tubules. Although the reduction in the percentage of KI67‐positive GFRα1‐expressing A_undiff_ is relatively modest in the presence of imatinib, over time, this could lead to a progressive decline in the mGSC pool size, resulting in smaller cohorts of differentiating germ cells and an increased probability of stochastic emptying of mGSC niche.^75^ Consequently, this may lead to a gradual reduction in spermatogenic output over time.

Another important finding in this study is that clinically relevant doses of imatinib, but not dasatinib, induced apoptosis in rat seminiferous tubules after 24 h exposure. Garcia et al. exposed male mice in vivo to a combination of dasatinib and flavonoid quercetin in 15 days over a 5‐month period. The treatment did not produce any observable effects on sperm motility, seminiferous tubule morphometry, testicular gene expression or fertility.^30^ Notably, the dasatinib and quercetin treatment resulted in an increased sperm concentration and a reduction in abnormal sperm morphology.^30^ Another study from the same authors reports that male mice receiving dasatinib and quercetin in vivo for 3 months did not have reduced sperm motility and fertility.^76^ Although these results are consistent with our observations regarding dasatinib, our findings are not entirely comparable since we did not include quercetin treatment in combination with dasatinib exposure.

The effects of imatinib on cultured mGSCs have also been investigated previously. While Heim et al. concluded that imatinib (1 µM) decreases the in vitro expansion of two independent cell lines of mGSCs, which is in line with our findings, enriched expression of differentiation markers c‐KIT and SOHLH1 (spermatogenesis and oogenesis specific basic helix‐loop‐helix 1) in cultured mGSCs make the results of Heim et al. challenging to interpret. They also reported that a subset of cultured cells (0.1%) was able to form spermatogenic colonies following transplantation into an infertile recipient, demonstrating the existence of stemness in the studied cell population.^77^ Altogether, these data suggest that their mGSC cultures consist of a mixture of stem and differentiating spermatogonia, which is in contrast to our mGSC cultures that are devoid of c‐KIT expression, making comparison of these two studies difficult.

Lack of c‐KIT expression is a demarcating feature of mGSCs. Given the fact that they are also devoid of expression of cancerous TK BCR‐ABL, and PDGFRs, which are important developmental regulators of the testis,^37^, ^38^, ^78^ the harmful effects of imatinib on mGSCs must be mediated via inhibition of alternative TKs. An intriguing candidate would be rearranged during transfection (c‐RET). c‐RET is a receptor tyrosine kinase that forms a complex with GFRα1 and functions as a receptor for GDNF, and is therefore critical for mGSC self‐renewal.^79^, ^80^ Interestingly, imatinib has also been implicated in the treatment of medullary thyroid carcinoma (MTC) which is associated with constitutively activated c‐RET.^81^, ^82^ Recently, more specific inhibitors of c‐RET have become available,^83^, ^84^ but those data nevertheless suggest that c‐RET is a potential imatinib target and reduction in proliferation of GFRα1‐positive A_undiff_ spermatogonia and cultured mGSCs might be mediated through inhibition of c‐RET tyrosine kinase.

While the effects of imatinib and dasatinib on mGSCs would require more mechanistic studies, the effects on differentiating germ cells can be justifiably explained by the inhibition of SCF/c‐KIT signaling, which has been shown to promote spermatogonial DNA synthesis and proliferation^32^, ^33^ and protect spermatogonia from apoptosis.^34^, ^63^, ^85^ We recorded dose‐dependently lower levels of ^3^H‐thymidine incorporation in stage V to VIII–IX cultured rat seminiferous tubule segments in the presence of imatinib, suggesting that imatinib interferes with S‐phase in both type B spermatogonia and preleptotene spermatocytes. This conclusion unfortunately has an obvious shortcoming because imatinib simultaneously also decreases the viability of these cells, and decreased DNA synthesis may be secondary to induction of apoptosis. While imatinib has been previously shown to inhibit DNA synthesis in primary human T cells,^62^ separating these two phenomena in our experimental model is very complicated. The ability of imatinib to induce apoptosis has been shown in numerous studies. ^62^, ^86^, ^87^, ^88^

Structural studies have shown that imatinib binding interferes with the inactive conformation of c‐KIT by inhibiting interaction of the autoinhibitory juxtamembrane domain with the kinase domains.^89^ Furthermore, in cancerous cells imatinib abolishes phosphorylation of a constitutively activated form of c‐KIT,^81^ leading to decreased proliferation and increased apoptosis in GIST cell lines.^90^, ^91^ While we presume that the effects of imatinib on c‐KIT expressed in the spermatogenic cells are mediated through interference of normal structural conformation c‐KIT, our data also imply an auxiliary mechanism. Notably, the adverse effects of imatinib on male germ cells might also be explained by decreased expression of c‐KIT, as suggested by our flow cytometry data. In cultured stage II–VI mouse seminiferous tubule segments, differentiation induction by RA was not disrupted per se in the presence of imatinib, as demonstrated by STRA8 expression in a vast majority of LIN28A‐positive A‐aligned spermatogonia. However, c‐KIT expression in these nascent differentiating spermatogonia was at a remarkably lower level in the presence of imatinib. While the underlying mechanisms would require further investigation, it is possible that blocking of c‐KIT signaling leads to decreased expression on *c‐Kit* mRNA, shorter lifespan of c‐KIT protein or removal of c‐KIT from the cell membrane. Whatever the mechanism, the outcome would be dampened c‐KIT signaling activity and poorer survival and proliferation of differentiating spermatogonia.

The imatinib doses used in this study (1–10 µM) correspond to mean plasma concentrations after clinically relevant single doses up to 1000 mg.^42^, ^43^, ^44^, ^45^, ^46^, ^47^ There is a significant variation in the plasma concentrations of imatinib between individuals and populations receiving cancer treatment.^44^ The mean plasma concentrations after a single dose regimen of 400 mg imatinib per day in Caucasian subjects are 1216.6 ng/mL (2.5 µM) with range of 121.2–4464.9 ng/mL (0.3–9.1 µM), whereas the mean plasma concentration in Asian subjects is higher at 1816.2 ng/mL (3.7 µM) with a range of 52.9–8257.3 ng/mL (0.1–16.7 µM). Only two clinical cases of imatinib overdose of 6400 and 16,000 mg have been reported.^49^, ^50^ Although not lethal, the estimated plasma concentration in these cases could be as much as 20 times higher compared to clinically relevant doses. Since imatinib plasma and semen concentrations correlate,^25^ in this study a 100‐µM dose was used to simulate the acute toxic effects of imatinib on the male germline.

The standard clinical dose of dasatinib is 100–180 mg, administered orally once daily. Plasma concentrations observed in patients typically range from 0.005 to 0.16 µM.^46^, ^48^, ^92^, ^93^ Clinical trials have indicated that optimal dasatinib plasma concentrations should be over 0.1 µM.^46^ However, concentrations as high as 0.8 µM have been detected in patients, as dosage adjustments are typically made to achieve target plasma concentrations, thereby enhancing efficacy and minimizing the risk of adverse events.^94^, ^95^, ^96^ A 10 µM dose of dasatinib was chosen to evaluate potential direct effects on male germ cells and mGSCs in experiments, where short exposure times were necessary due to technical limitations. A dose leading to 10 µM dasatinib concentration has been evaluated in rats in vivo during 10 days. This dose exhibited potent therapeutic activity and a high safety margin in a CML animal model. Importantly, this dosage did not result in toxicity, as evidenced by the absence of animal mortality or impaired weight gain.^17^

Previous studies have shown that imatinib‐treated male patients have reduced sperm counts.^26^, ^78^ We address here what might explain these findings using adult rat and mouse models and ex vivo and in vitro culture techniques. Our observations suggest that the decreased sperm counts in imatinib‐treated male patients may be due to decreased mGSC proliferation and interference of SCF/c‐KIT signaling in spermatogenic cells, leading to smaller numbers of differentiating germ cells, gradual decline in the number of stem cells, lower number of proliferating spermatogonia and germ cell apoptosis. This study provides plausible explanations for how imatinib might disturb spermatogenic potential in patients and why dasatinib might have lesser impact on spermatogenesis. Since the outcomes observed with dasatinib differ from those seen with imatinib, the results of one TKI may not be generalizable to effects of all of them. Our findings suggest that caution is advised when considering the use of imatinib and dasatinib in young patients beyond oncology.

## 5 CONCLUSIONS

In this study, we investigated the effects of first and second‐generation TKIs imatinib and dasatinib on adult rat spermatogenic cells and mouse mGSCs using ex vivo and in vitro culture. Our results indicate that clinically relevant doses of imatinib, but not dasatinib, increase germ cell apoptosis, and clinical doses of both drugs compromise the self‐renewal of mGSCs in vitro. Our data indicate that imatinib's pro‐apoptotic effect on differentiating germ cells is partly due to the down‐regulation of c‐KIT expression and inhibition of SCF/c‐KIT signaling.

## AUTHOR CONTRIBUTIONS

Anna Eggert and Juho‐Antti Mäkelä were responsible for writing the manuscript, analyzing the data and carrying out the majority of the experiments. Kirsi Jahnukainen, Jorma Toppari, Mirja Nurmio, and Aida Wahlgren participated in the beginning experimental design. Mirja Nurmio and Aida Wahlgren performed the first experiments. Anna Eggert, Juho‐Antti Mäkelä, Jorma Toppari, and Noora Kotaja contributed to the final experimental design. Anna Eggert, Sini Laasanen, Miisael Nieminen, and Opeyemi Olotu performed the final experiments. Kim Eerola participated in the writing process. Jorma Toppari, Noora Kotaja, and Kirsi Jahnukainen acquired funding. All reviewed, commented and approved.

## CONFLICT OF INTEREST STATEMENT

The authors declare no conflicts of interest.

## Supporting information



Supporting information

Supporting information

Supporting information

Supporting information

Supporting information

Supporting information

Supporting information

Supporting information

## Data Availability

The data that support the findings of this study are available from the corresponding author upon reasonable request.
