# Supplementary material for: Imatinib decreases germ cell survival and germline stem cell proliferation in rodent testis ex vivo and in vitro
Source: Andrology. 2024 Oct 18;13(6):1575–91. doi: 10.1111/andr.13777 (PMC12368934; doi:10.1111/andr.13777)
Supplement: Supplementary file 1 — Supporting information [file ANDR-13-1575-s006.pdf]

SUPPLEMENTAL FIGURE 1

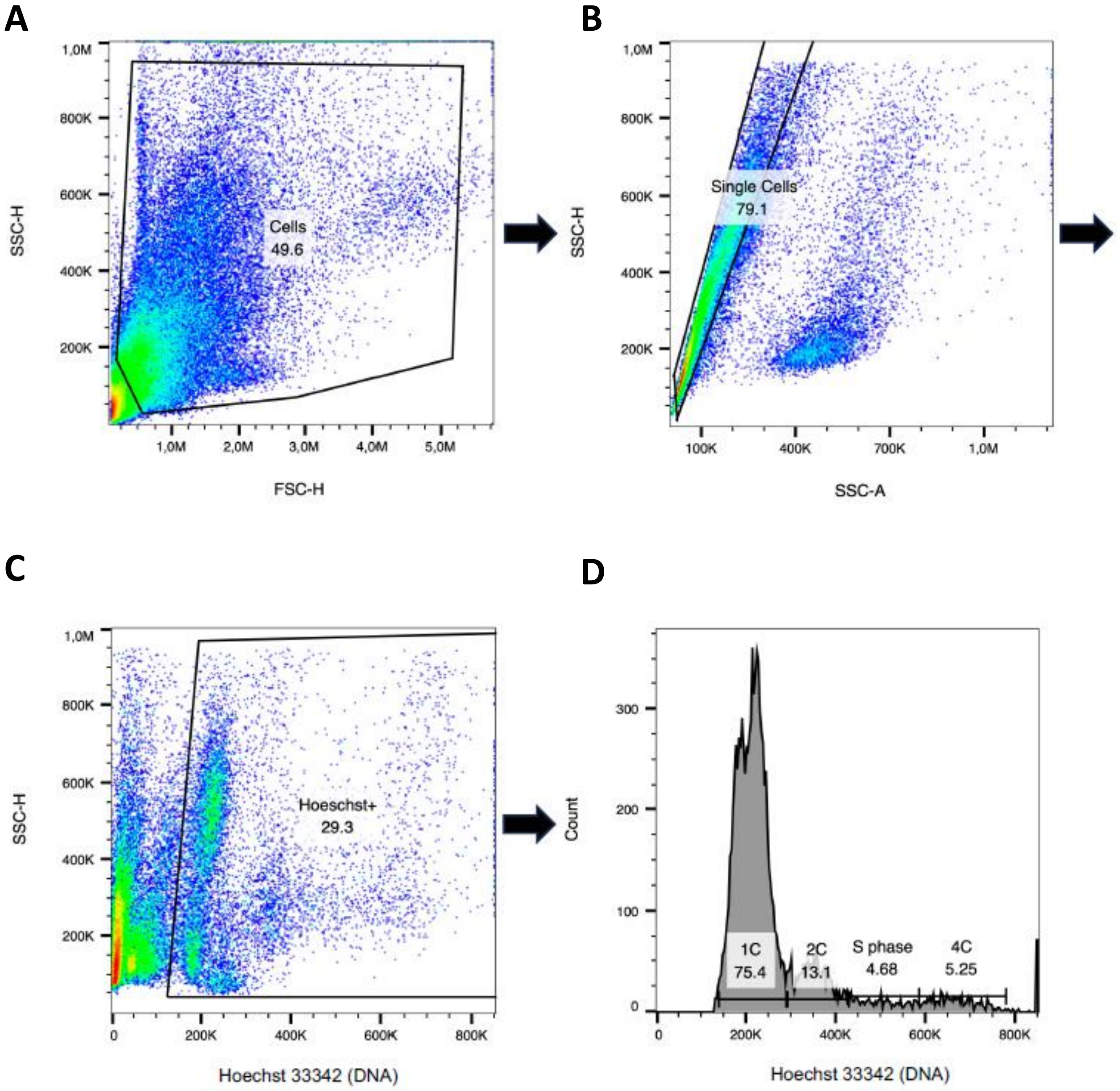

SUPPLEMENTAL FIGURE 1. Gating strategy for flow cytometry analysis following Hoechst 33342 staining for single-cell suspensions prepared from cultured stage VII-VIII seminiferous tubule segments.
