# Supplementary material for: Imatinib decreases germ cell survival and germline stem cell proliferation in rodent testis ex vivo and in vitro
Source: Andrology. 2024 Oct 18;13(6):1575–91. doi: 10.1111/andr.13777 (PMC12368934; doi:10.1111/andr.13777)
Supplement: Supplementary file 2 — Supporting information [file ANDR-13-1575-s005.pdf]

SUPPLEMENTAL FIGURE 2

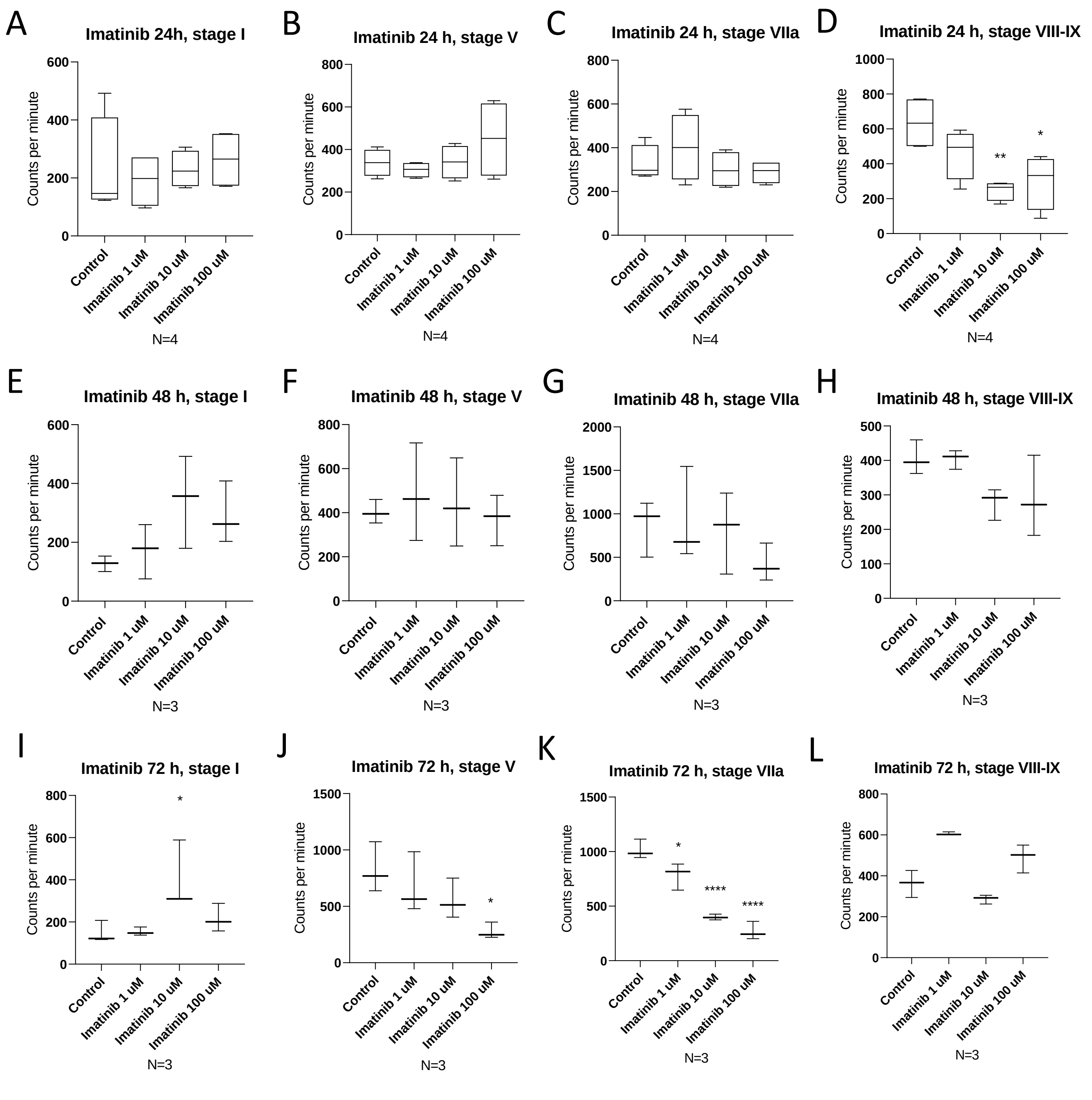

SUPPLEMENTAL FIGURE 2. ^{3}H-thymidine incorporation in cultured rat seminiferous tubule segments. (A) – (L) Seminiferous tubule segments of stages I, V, VIIa and VIII-IX were cultured for 24 h (n=4), 48 h (n=3) and 72 h (n=3) in the absence or presence of imatinib (IM) (1, 10 and 100 µM) followed by measurement of ^{3}H-thymidine incorporation to study the amount of DNA-synthesis during culture. One-way ANOVA, followed by Tukeys´s multiple comparison test. ****$p < 0.0001$, *** $p < 0.001$, ** $p < 0.01$, *$p < 0.05$
