# Supplementary material for: Imatinib decreases germ cell survival and germline stem cell proliferation in rodent testis ex vivo and in vitro
Source: Andrology. 2024 Oct 18;13(6):1575–91. doi: 10.1111/andr.13777 (PMC12368934; doi:10.1111/andr.13777)
Supplement: Supplementary file 3 — Supporting information [file ANDR-13-1575-s002.pdf]

SUPPLEMENTAL FIGURE 3

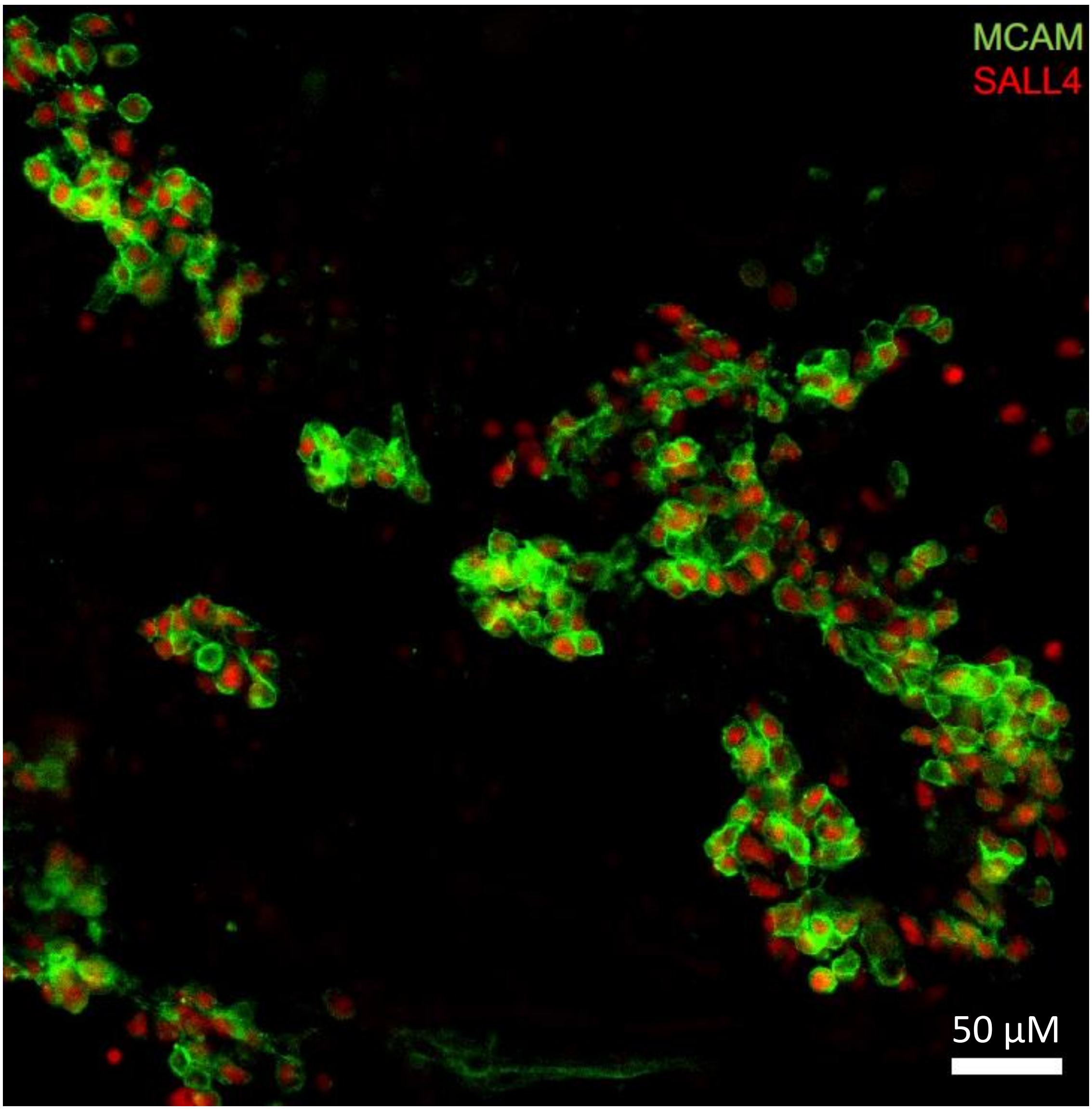

SUPPLEMENTAL FIGURE 3. mGSC colonies express MCAM and SALL4. The identity of mGSCs in culture was confirmed by their grape-like colony morphology and staining for MCAM (green; melanocyte cell adhesion molecule[65]) and SALL4 (red; Spalt-like 4[66]) expression. Scalebars 50 µm.
