# Supplementary material for: Imatinib decreases germ cell survival and germline stem cell proliferation in rodent testis ex vivo and in vitro
Source: Andrology. 2024 Oct 18;13(6):1575–91. doi: 10.1111/andr.13777 (PMC12368934; doi:10.1111/andr.13777)
Supplement: Supplementary file 4 — Supporting information [file ANDR-13-1575-s008.pdf]

SUPPLEMENTAL FIGURE 4

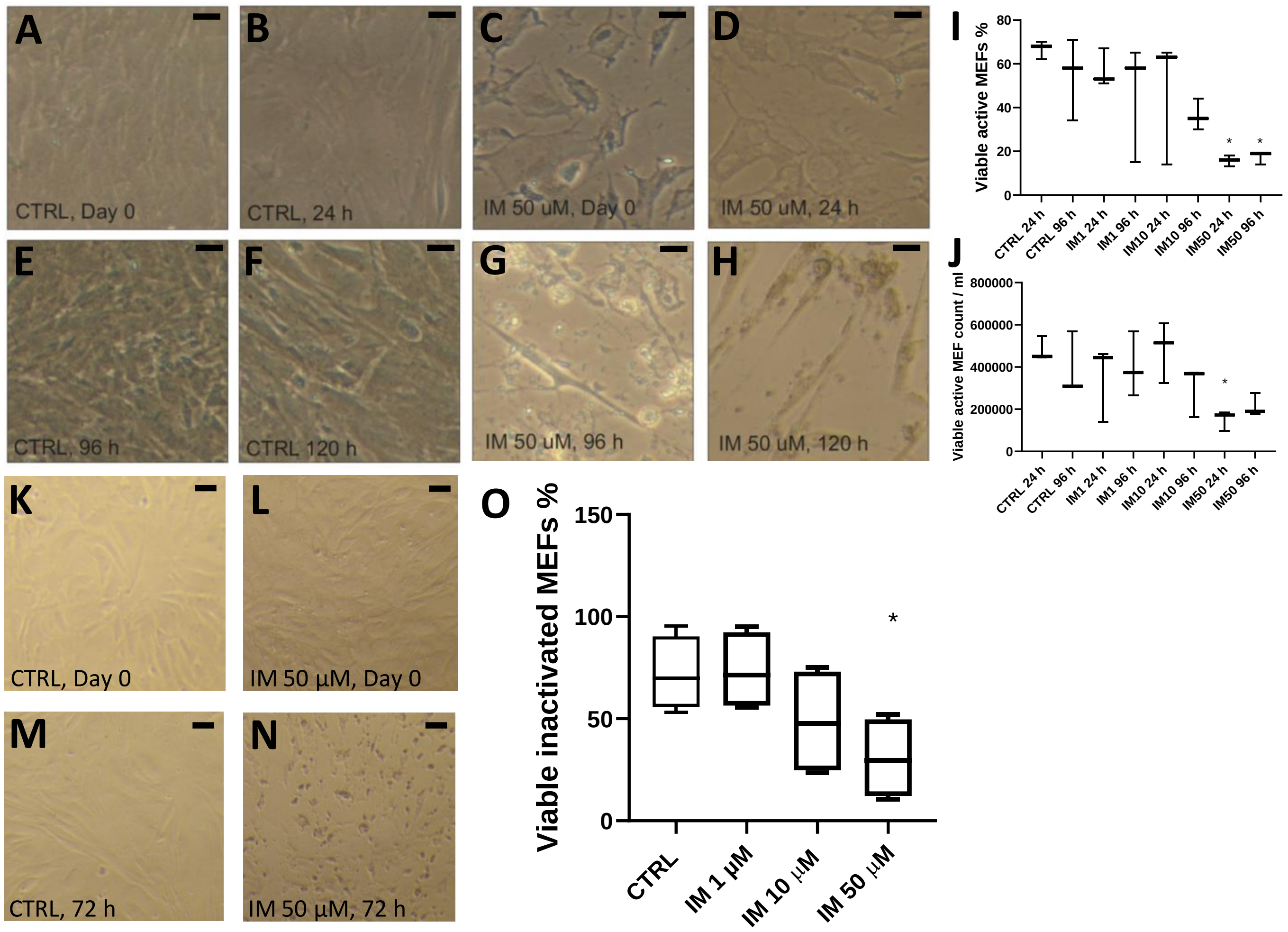

SUPPLEMENTAL FIGURE 4. Imatinib did not have an adverse effect on MEFs at doses of 1-10 µM In order to find an optimal imatinib dose for mGSC culture enabling the direct effects to be studied, imatinib exposure on MEFs was done before mitomycin-inactivation in three different doses and different exposure times: 1 µM, 10 µM and 50 µM and 24 h, 96 h and 120 h (A) – (J). Representative images of control and IM 50 µM show the MEF layers condition before (Day 0) and after the specific exposure time. The effects of imatinib on MEFs was also studied (K) – (O) after mitomycin-inactivation for 72 h. Trypan Blue was used to assess cell viability. n=4. One-way ANOVA followed by Dunnett´s multiple comparison test. Median, +/- max and min. $*p < 0.05$. Scalebars 50 µm.
