# Supplementary material for: Imatinib decreases germ cell survival and germline stem cell proliferation in rodent testis ex vivo and in vitro
Source: Andrology. 2024 Oct 18;13(6):1575–91. doi: 10.1111/andr.13777 (PMC12368934; doi:10.1111/andr.13777)
Supplement: Supplementary file 5 — Supporting information [file ANDR-13-1575-s003.pdf]

SUPPLEMENTAL FIGURE 5

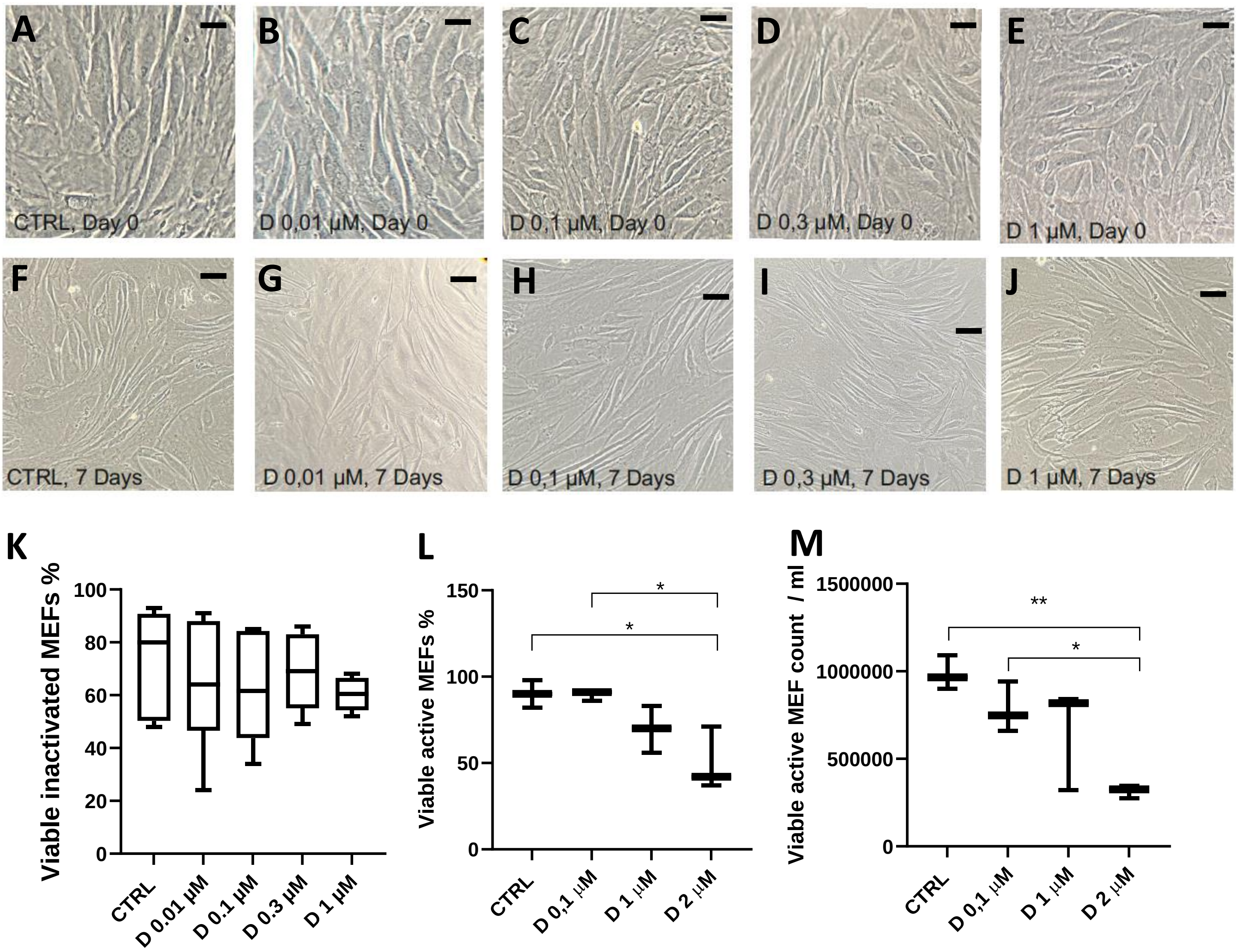

SUPPLEMENTAL FIGURE 5. Dasatinib did not have adverse effect on MEFs at doses 0.01-1 µM. Likewise, in order to find an optimal dasatinib dose for mGSC culture, (A) – (K) dasatinib 1 week exposure on MEFs after mitomycin-inactivation was done in five different doses: 0, 0.01 µM, 0.1 µM, 0.3 µM and 1 µM. (L) – (M) Also dasatinib 72 h exposure on MEFs before mitomycin-inactivaton was done in doses 0.1 - 2 µM. Trypan blue was used to assess cell viability. n=6 (inactivated MEFs) and n=3 (active MEFs). One-way ANOVA followed by Dunnett´s multiple comparison test. Median, +/- max and min. *$p < 0.05$. Scalebars 50 µm.
