# Supplementary material for: Imatinib decreases germ cell survival and germline stem cell proliferation in rodent testis ex vivo and in vitro
Source: Andrology. 2024 Oct 18;13(6):1575–91. doi: 10.1111/andr.13777 (PMC12368934; doi:10.1111/andr.13777)
Supplement: Supplementary file 6 — Supporting information [file ANDR-13-1575-s007.pdf]

SUPPLEMENTAL FIGURE 6

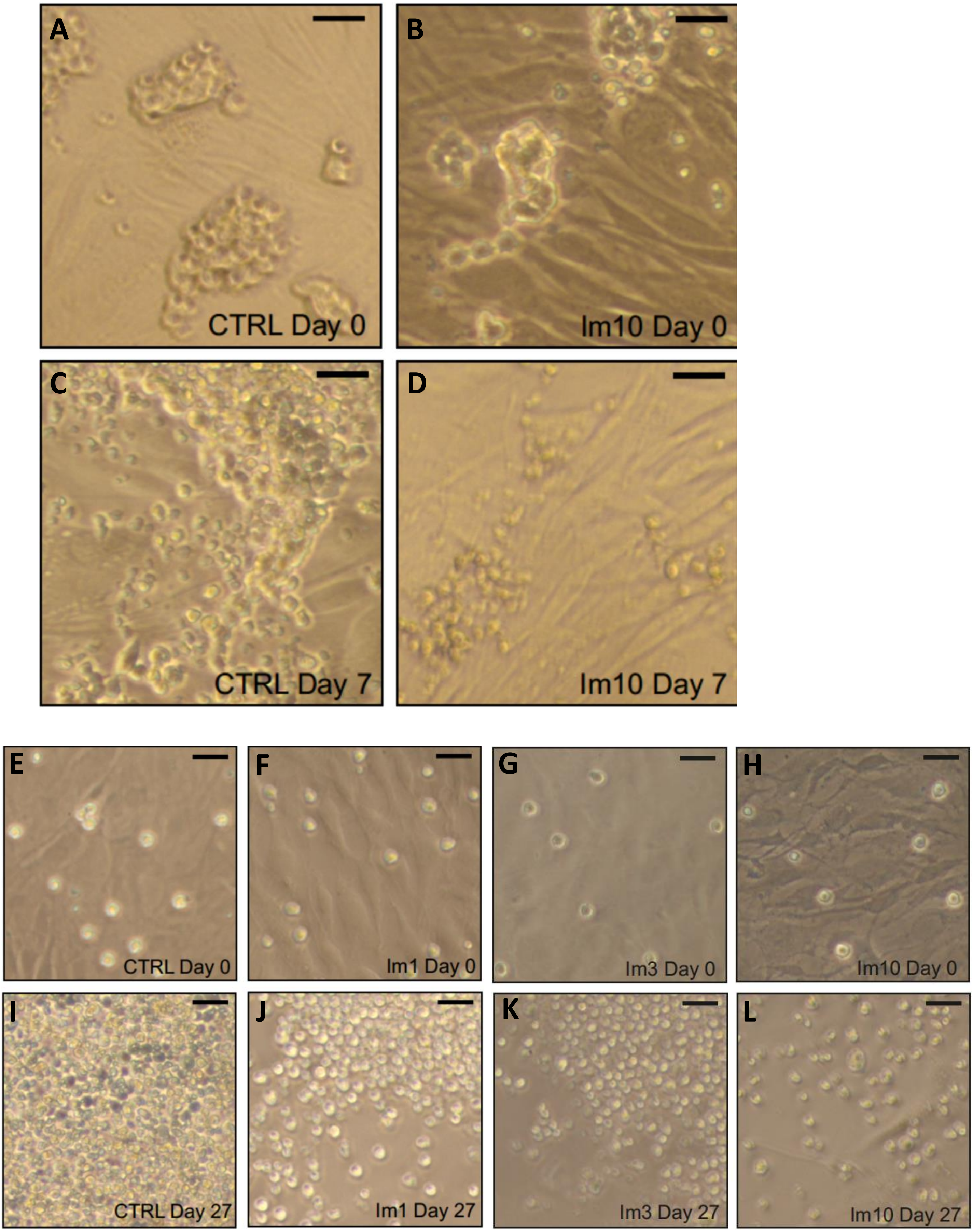

SUPPLEMENTAL FIGURE 6. Morphology of mGSCs in culture in the presence or absence of imatinib. mGSCs were cultured and exposed to imatinib using two different experimental setups. (A) – (D) mGSCs were cultured in the absence of imatinib until they formed small colonies and imatinib (0, 1, 5 and 10 µM) was then applied for 7 days. (E) – (L) Imatinib exposure on mGSCs was started following passaging and the cells were cultured for 4 weeks in the absence or presence of imatinib (0, 1, 3 and 10 µM). Scalebars 50 µm.
