# Supplementary material for: Imatinib decreases germ cell survival and germline stem cell proliferation in rodent testis ex vivo and in vitro
Source: Andrology. 2024 Oct 18;13(6):1575–91. doi: 10.1111/andr.13777 (PMC12368934; doi:10.1111/andr.13777)
Supplement: Supplementary file 7 — Supporting information [file ANDR-13-1575-s004.pdf]

SUPPLEMENTAL FIGURE 7

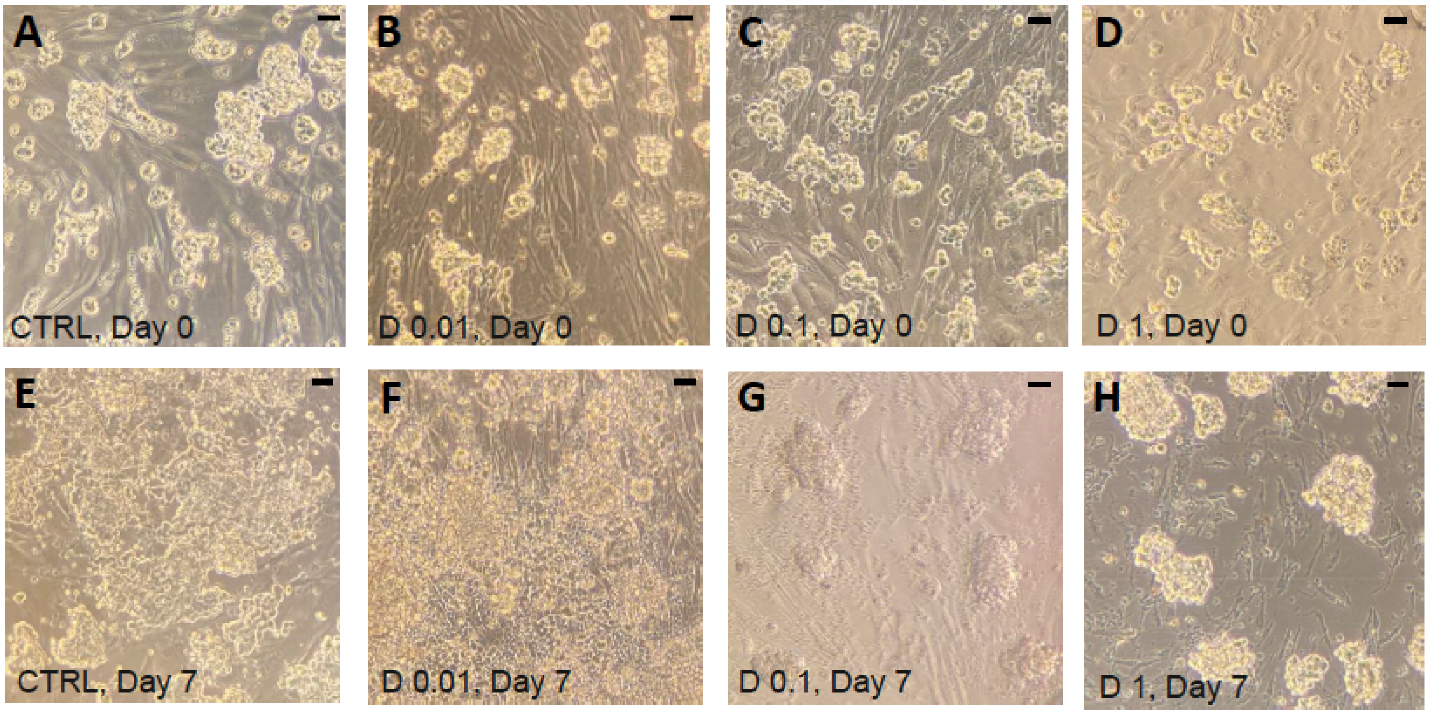

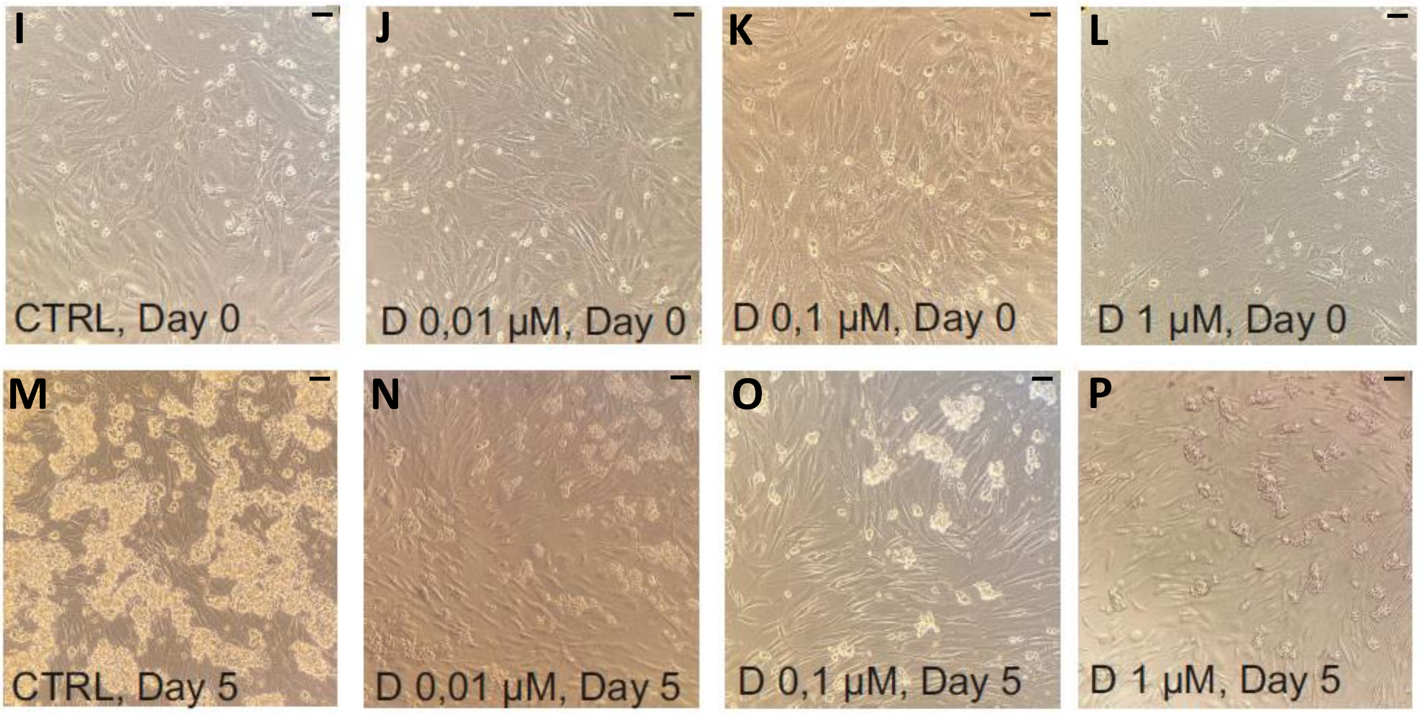

SUPPLEMENTAL FIGURE 7. Morphology of mGSCs in culture in the presence or absence of dasatinib. mGSCs were cultured and exposed to dasatinib using two different experimental setups. (A) – (H) mGSCs were cultured in the absence of dasatinib until they formed small colonies and dasatinib (0, 0.01, 0.1, 0.3 and 1 µM) was then applied for 7 days. (I) – (P) Dasatinib exposure on mGSCs was started following passaging and the cells were cultured for 5 days in the absence or presence of dasatinib (0, 0.01, 0.1, 1, 0.3 and 1 µM). Scalebars 50 µm.
