# Supplementary material for: Imatinib decreases germ cell survival and germline stem cell proliferation in rodent testis ex vivo and in vitro
Source: Andrology. 2024 Oct 18;13(6):1575–91. doi: 10.1111/andr.13777 (PMC12368934; doi:10.1111/andr.13777)
Supplement: Supplementary file 8 — Supporting information [file ANDR-13-1575-s001.pdf]

**SUPPLEMENTAL FIGURE 8**

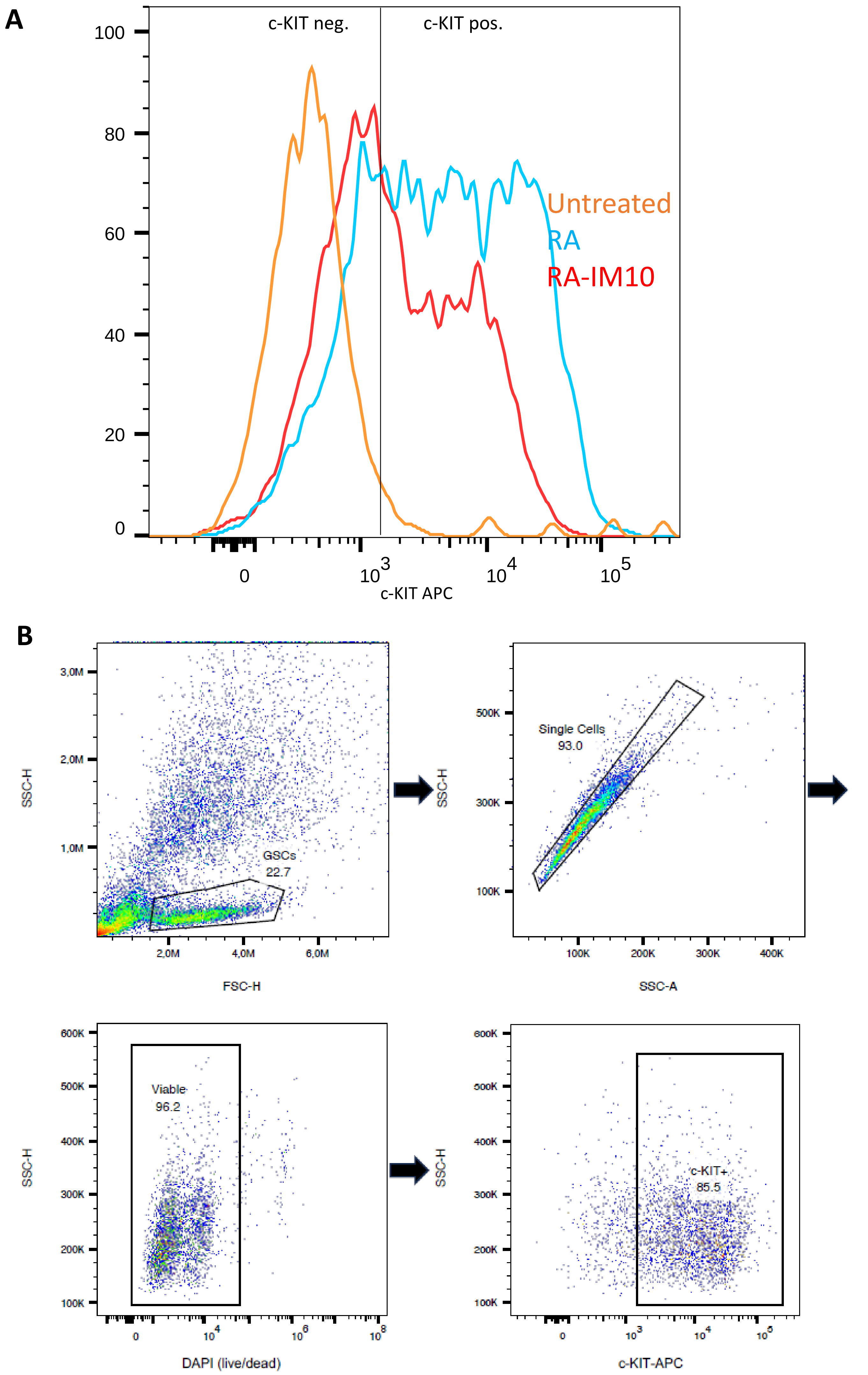


**SUPPLEMENTAL FIGURE 8. Attenuated levels c-KIT expression induction in imatinib-treated mGSCs cultures following RA treatment.** (A) Representative histograms showing the distribution of c-KIT-APC signal in untreated (orange), RA-treated (blue) and RA+IM10-treated cultured mGSC (red). (B) Gating strategy for flow cytometry analysis of c-KIT expression in mGSC cultures. An RA-treated sample is shown.
